# Re-thinking reablement strategies for older adults in residential aged care: a scoping review

**DOI:** 10.1186/s12877-021-02627-7

**Published:** 2021-11-30

**Authors:** Lucy K. Lewis, Tim Henwood, Jo Boylan, Sarah Hunter, Belinda Lange, Michael Lawless, Rachel Milte, Jasmine Petersen

**Affiliations:** 1 Caring Futures Institute, College of Nursing and Health Sciences, Flinders University, GPO Box 2100, Adelaide, SA 5001 Australia; 2 Southern Cross Care (SA, NT & Vic) Inc., PO Box 155, Glen Osmond, SA 5064 Australia

**Keywords:** Reablement strategies, Older adults, Aged care, Physical deconditioning, Feasibility, Acceptability, Cost effectiveness

## Abstract

**Background:**

The number of older adults in residential aged care is increasing. Aged care residents have been shown to spend most of the day sedentary and have many co-morbidities. This review aimed to systematically explore the effectiveness of reablement strategies in residential aged care for older adults’ physical function, quality of life and mental health, the features of effective interventions and feasibility (compliance, acceptability, adverse events and cost effectiveness).

**Method:**

This scoping review was undertaken according to PRISMA guidelines (extension for scoping reviews). Five e-databases (Medline, Embase, Cochrane Central Register of Controlled Trials, Cochrane Database of Systematic Reviews and CINAHL) were searched from 2010 onwards. Randomised controlled trials investigating reablement strategies addressing physical deconditioning for older adults (mean age ≥ 65 yrs) in residential aged care on physical function, quality of life or mental health were included. Feasibility of the interventions (compliance, acceptability, satisfaction, adverse events and cost effectiveness) was explored.

**Results:**

Five thousand six hundred thirty-one citations were retrieved, and 63 studies included. Sample sizes ranged from 15 to 322 and intervention duration from one to 12 months. Exercise sessions were most often conducted two to three times per week (44 studies) and physiotherapist-led (27 studies). Interventions were predominately multi-component (28 studies, combinations of strength, balance, aerobic, functional exercises). Five interventions used technology. 60% of studies measuring physical function reported significant improvement in the intervention versus control, 40% of studies measuring quality of life reported significant improvements in favour of the intervention, and 26% of studies measuring mental health reported significant intervention benefits. Over half of the studies measured compliance and adverse events, four measured acceptability and none reported cost effectiveness.

**Conclusions:**

There has been a research surge investigating reablement strategies in residential aged care with wide variability in the types and features of strategies and outcome measures. Few studies have measured acceptability, or cost effectiveness. Exploration of core outcomes, mapping stakeholders and co-designing a scalable intervention is warranted.

**Trial registration:**

Prospectively registered review protocol (Open Science Framework: DOI 10.17605/OSF.IO/7NX9M).

**Supplementary Information:**

The online version contains supplementary material available at 10.1186/s12877-021-02627-7.

## Background

Of the 1.2 million Australians receiving aged care services in Australia, greater than 270,000 reside in residential aged care (RAC), representing the greatest proportion of government aged care spending [1]. The aged care sector is expanding to match the growing older Australian population with 125 aged care places to be provided per 1000 people aged 70 years or older in 2021-2, with most of these in RAC [1]. Concerns continue to be raised about the quality of life (QOL) of many older adults living in RAC, particularly those living with cognitive or functional impairment, highlighting an urgent and persistent problem. Older Australians in RAC are at high risk of physical deconditioning due to their age, functional impairment, complex co-morbidities and sedentary behaviour [2]. Physical deconditioning is the physiological change from illness, disease or inactivity that results in decreased muscle mass, weakness, functional decline, and difficulty performing daily living activities [3, 4]. Physical deconditioning is a hallmark of frailty, an age-associated clinical syndrome reflecting decreased physiological reserves and increased susceptibility to the impacts of external stressors [5, 6]. It is estimated that approximately 2.1 million Australians aged 65 and over are pre-frail or frail [7], with this number set to increase rapidly in the coming decade.

Exercise has been shown to be an effective countermeasure to physical deconditioning among RAC adults [8]. The benefits extend past falls prevention and increased physical and mental wellbeing, to having significant positive financial implications [9]. Despite this evidence, there are inconsistencies in the levels of support RAC facilities provide for reablement pathways of residents. While there is a growing body of evidence supporting the benefits of exercise for older people in RAC, there are a number of challenges for aged care organisations. Potential barriers include resourcing for restorative exercise and social activities, organisational culture, funding models, and knowledge, experience and skills of staff and residents [10].

Programs aimed at promoting physical reconditioning have the potential to improve residents’ wellbeing and align to current aged care standards. A recently commissioned review for the Australian Royal Commission into Aged Care Quality and Safety investigating innovative models of care advocated a ‘wellness and reablement’ approach in RAC, where people are assisted to regain functional capacity and improve independence through innovative models of care including exercise, activities of daily living (ADL) retraining, and behavioural interventions [11]. Reablement is a term used generally to describe a targeted, time-limited approach that promotes the regaining or maintenance of functional performance. Reablement specifically in aged care refers to programs that help individuals re-establish daily living skills through goal-oriented programs [12, 13]. A previous systematic review [14] investigated and evaluated the benefits and harms of rehabilitation interventions to maintain or improve physical function for older adults in long-term care. The authors concluded that physical rehabilitation may be effective, but there was insufficient evidence to make conclusions about sustainability, cost-effectiveness or the most appropriate intervention types. Therefore, our review aimed to scope the current evidence for reablement strategies for older adults in RAC, including exploration of effectiveness (physical function, QOL, mental health), features of effective interventions, and feasibility (compliance, acceptability, adverse events and cost effectiveness).

## Methods

A scoping review of the literature was undertaken according to the PRISMA guidelines (extension for scoping reviews) [15] to explore gaps in the existing evidence in the broad area of reablement strategies for older people in RAC [16]. Scoping review methodology was most suited to the exploration of the broader aims of the review, including descriptive exploration of effectiveness of multiple outcomes, features of effective interventions and feasibility. The protocol was prospectively registered with the Open Science Framework, DOI 10.17605/OSF.IO/7NX9M (https://osf.io/7nx9m/).

### Identification and selection of studies

#### Search strategy

Five databases (Medline, Embase, Cochrane Central Register of Controlled Trials, Cochrane Database of Systematic Reviews and CINAHL) were searched (August 2020) using both subject heading and keyword searches where possible. The search strategy was peer-reviewed by an academic librarian and is included in e-Addenda (Additional file 1).

#### Eligibility criteria

A previous Cochrane systematic review provided a comprehensive overview of RCTs reporting interventions aimed at maintaining or improving physical function in older people in long-term care [14]. Therefore, to be included, studies must have included original primary data and have a randomised controlled trial (RCT) design at the participant level. The search for this previous review was completed at the end of 2009, therefore the current review focussed on 2010 onwards. The inclusion criteria for the review are summarised in Table 1.

**Table 1:** Inclusion criteria

*Design*	
• Randomised controlled trial	
*Participants*	
• Mean age ≥ 65 years old	
• Permanent aged care resident	
*Intervention*	
• Must address physical deconditioning and aim to maintain or improve physical function	
• Must have involved the participants themselves	
*Outcome measures*	
• Physical function	
• Quality of life	
• Mental health	
• Feasibility	
*Comparator/s*	
• Another intervention aimed at improving physical function	
• No intervention / placebo	

To be included, studies must have included older people (mean age ≥ 65 years) residing in a RAC facility as their permanent place of abode. Residential aged care was defined as an institutional setting where care is provided for older people 24 h a day, 7 days a week, including assistance with ADLs. Studies with participants residing in short- or long-term medical facilities or retirement accommodation with minimal or no assistance available were excluded.

The intervention was not required to be labelled as reablement or restorative care but must have aimed to maintain or improve physical function, and include a targeted and goal-oriented approach, consistent with the definitions of reablement in the aged care setting [12, 13]. This typically involved active movement and was in the form of specific exercises, or physical activity as part of another activity. The intervention must have involved the participants themselves, rather than being solely targeted at the physical environment or setting.

For inclusion, studies must have included at least one outcome of interest (physical function, QOL or mental health) and report between group analyses for these outcome/s. Physical function may have been measured with an independence scale such as the Barthel Index, Functional Independence Measure (FIM), Short Physical Performance Battery (SPPB) or tests of ability in ADLs such as mobility or transfers (e.g. Timed Up and Go (TUG), 6-m walk test (6MWT)). Quality of life must have been measured by a validated self-reported tool such as the Life Satisfaction Index, 12-item Short Form Survey (SF-12) of the 36-item Short Form Survey (SF-36) measures of Health-related QOL or the General Well-being Scale. Mental health measures may have included measures of mood, depression, or anxiety, for example, the State-Trait Anxiety Inventory (STAI), Beck Depression Inventory (BDI), or the Geriatric Depression Scale (GDS). Feasibility measures (e.g. satisfaction, cost, burden, adherence, adverse effects) related to the intervention were extracted. If reference was made to a previous published study with information regarding intervention development or feasibility measures, this was noted, and the reference retrieved.

Studies were included that compared an intervention aimed at physical function with either no intervention / placebo, or an alternative intervention.

#### Procedure

Citations were extracted from the electronic databases into the Covidence online platform (https://www.covidence.org/) and duplicates removed. Two independent reviewers (LKL, JP) completed two rounds of screening for the review. First, titles and abstracts were screened against the eligibility criteria. Disagreements were resolved by discussion, with a third independent reviewer (RM). Citations with no abstract available or where ambiguity existed were retained. The second round of screening full text citations was completed by two independent reviewers (LKL, JP), with disagreements resolved by discussion with a third independent reviewer (RM). Relevant systematic reviews were retained, and the reference lists searched. The reference lists of included studies were screened for further eligible studies.

### Assessment of characteristics of studies

Scoping reviews can be differentiated from systematic reviews in that they are generally conducted to provide an overview of the available evidence, regardless of methodological quality or risk of bias [15]. As the broad aim of this review was to descriptively explore reablement strategies in RAC facilities, critical appraisal of the included studies was not completed in line with scoping review methodology [16, 17].

### Data analysis

Two reviewers (LKL, JP) independently completed data extraction of the first 20% of included studies, and then met to compare extracted data. Following this, adjustments were made to the data extraction template, and the remainder of the extraction was completed by one reviewer (JP) and cross-checked by a second reviewer (LKL). A narrative synthesis was performed in line with the scoping review questions and aims.

## Results

### Flow of studies through the review

**Fig. 1:**
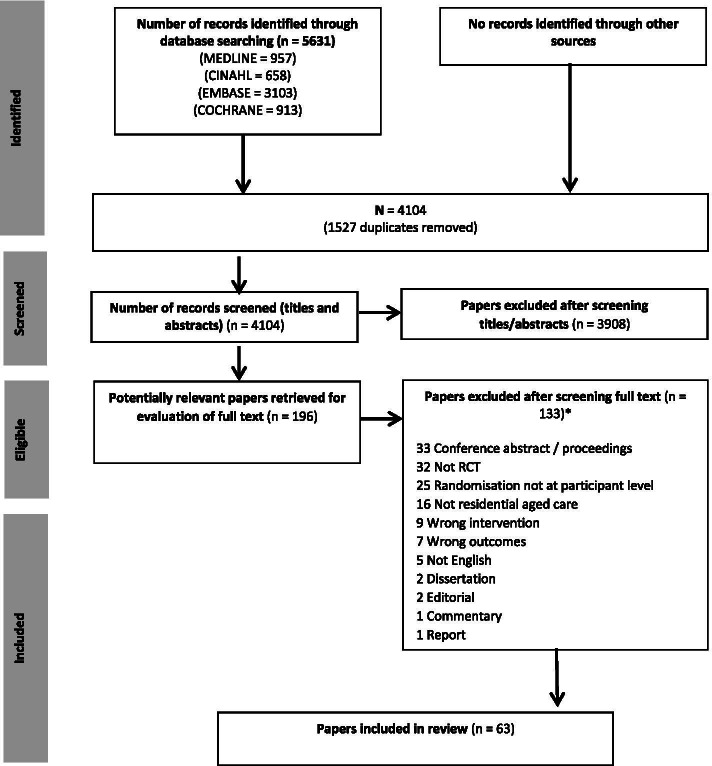
Flow of studies through review *Papers may have been excluded for failing to meet more than one inclusion criteria. The primary exclusion criterion that had consensus among reviewers was recorded and used for reporting

A total of 5631 citations were retrieved from the search of electronic databases (Fig. 1). Following removal of duplicates, 4104 titles and abstracts were screened, with 3908 excluded. One-hundred and ninety-six full text papers were screened, with 133 of these excluded. The most common exclusion reasons were conference abstracts and proceedings (*n* = 33), not RCT (*n* = 32) and randomisation not at the individual participant level (*n* = 25). A final 63 studies were included in the scoping review (see Additional file 2 for a detailed summary of included studies).

### Characteristics of studies

Studies were published between 2010 and 2020, with 38% (*n* = 24) of the included studies published between 2018 and 2020. Forty-six studies were conducted in Europe [18–63], eight in America [43, 64–70], seven in Asia [71–77], one in Australia [78], and one in New Zealand [79]. None of the included studies referenced a previous study which reported intervention development, process design or feasibility measures relating to the interventions.

#### Participants

Baseline sample sizes ranged from 15 [50] to 322 participants [35, 36]. Over half of the included studies (*n* = 33) reported a power calculation. Participants were residents of nursing homes (*n* = 36), residential care homes/facilities (*n* = 12), long-term care homes/facilities (*n* = 10), assisted living facilities (*n* = 4) and institutions (*n* = 3). The mean age of participants was 82.7 (6.9) years, and on average 70% were female. Almost half of the included studies (*n* = 30) studied specific subpopulations, including those with; dementia [25, 29, 52–54, 66, 73], Alzheimer’s disease [63, 69], cognitive impairment [29, 41, 64, 76], depressive symptoms [42], sarcopenia [38], osteopenia and osteoporosis [45], impaired ambulatory function [72, 75], low physical function [50], or those who were frail [27, 30, 48, 60, 65, 79], dependent in ADLs [32, 35, 36], sedentary [61] or used wheelchairs for mobility [71, 77].

#### Interventions

Intervention durations ranged from one [73, 75] to 12 months [29, 59], and were predominately 12 (*n* = 24), 24 (*n* = 7) or 16 weeks (*n* = 6). Follow-up assessments were conducted in 27% of included studies at the following timeframes: 12 weeks (*n* = 2), 18 weeks (*n* = 1), 6 months (*n* = 8), 7 months (*n* = 2), 10 months (*n* = 1), 12 months (*n* = 2), and 16 months (*n* = 1). Exercise session frequency ranged from one [31, 37, 43, 57] to seven times [29, 35, 36, 59] per week, with sessions most commonly conducted two (*n* = 20) or three times (*n* = 24) per week. The duration of individual sessions ranged from 10 [64] to 80 min [50]. The exercise sessions were commonly delivered and/or supervised by physiotherapists / physical therapists (*n* = 27), professional instructors (e.g., Tai Chi, dance) (*n* = 10), research personnel (*n* = 8), exercise therapists/specialists, physical trainers (*n* = 6), occupational therapists (*n* = 4), staff members (*n* = 4) and kinesiologists (*n* = 2).

The interventions were predominately multicomponent (*n* = 28), specifically, they most commonly incorporated various combinations of the following types of exercises; strength (*n* = 26), balance (*n* = 21), aerobic (*n* = 17), and functional (*n* = 6). Seven interventions exclusively incorporated strength training [23, 30, 38, 39, 56, 60, 73] and six exclusively incorporated aerobic training, in particular, walking [63, 64, 66] and cycling [29, 58, 59]. The interventions also incorporated Tai Chi (*n* = 4) [33, 71, 77, 78], dance (*n* = 3) [37, 43, 57], and exercise with ‘Whole Body Vibration’ (*n* = 7) [18, 22, 26, 50, 51, 72, 79]. Four interventions were delivered using interactive game-based technologies, including commercially available video-game technologies (Nintendo Wii-Fit [69], Microsoft Xbox-360 [70]), and interactive camera-based technologies (BTS NIRVANA system [62] and Jintronix [67]). One intervention involved a digital sports watch to monitor and provide prompts for regular activity [64].

Overall, 38% of studies (*n* = 24) provided information pertaining to intervention development. Interventions were developed in consultation with physiotherapists [30, 41], occupational therapists [31], physical activity professionals [30], and experts and practitioners for physical activity groups in aged care [31]. Interventions were also based on; previously developed interventions [32, 40, 43, 46, 47, 52–54, 57, 71, 77], existing literature or recommendations [19–21, 24, 31, 42, 73, 80] and the needs and preferences of participants [24, 35, 36, 65].

#### Comparators

Fifty studies (79%) compared two groups; the physical activity intervention was compared to a control group that engaged in either usual care (*n* = 31), a social or recreational activity (*n* = 8), or a different program including physical activity (*n* = 11). Nine studies (14%) compared three groups, specifically, two physical activity programs were compared with a control group (usual behaviour, social/ recreational activity) [25, 33, 38, 48, 58, 72, 78, 79], and three physical activity programs were compared [55]. Several studies compared four groups; three physical activity programs compared with a control [23, 45, 80] and a comparison of two physical activity programs with and without verbal stimulation [61].

#### Outcome measures

Fifty-five (88%) included studies measured and reported physical function. Measures that were used in multiple studies include the TUG test (*n* = 38), Repeated Chair Stand test (30-s chair stand or 5-time sit-to-stand) (*n* = 21), gait speed (*n* = 18) with distances ranging from four [20] to 800 [70] metres, Berg Balance Scale (*n* = 17), SPPB (*n* = 10), gait distance (e.g., 6MWT) (*n* = 7), Tinetti test (*n* = 6), Chair Sit and Reach test (*n* = 4), Senior Fitness test (*n* = 4), physical activity (accelerometer-derived) (*n* = 3), walking/wheel chair propulsion (*n* = 2), 2 min step test (*n* = 2), Nursing Home Life Space Diameter (*n* = 2), Clinical Outcome Variables Scale (*n* = 2), Study Osteoporotic Fractures Index (frailty) (*n* = 2), Frailty and Injuries Cooperative Studies of Intervention Techniques (*n* = 2), and the Fried Frailty Phenotype (*n* = 2). Measures of ADLs included the Barthel Index (*n* = 17), Katz Index (*n* = 6), Functional Independence Measure (*n* = 4), Lawton IADL scale (*n* = 3), Physical Performance test (ADLs and IADLs) (*n* = 1), and Canadian Occupational Performance Measure (*n* = 1).

Twenty studies (32%) included QOL as an outcome. Measures used to assess QOL were Quality of Life in Alzheimer’s Disease Scale [19, 46, 47, 69], SF-36 [28, 42, 61, 64, 67], EQ-5D [18, 31], WHOQOL-BREF [48, 70, 71], Quality of Life in Late Stage Dementia Scale [52, 53], SF-12 [24], Satisfaction with Life Scale [24], WHOQOL-OLD [48], QUALEFO-41 [45], Dementia QOL [78], Life Satisfaction Index [74], and 15D-QOL [55].

Nineteen studies (30%) assessed mental health outcomes, largely anxiety and depression. Measures used to assess mental health were the Geriatric Depression Scale [28, 32, 33, 48, 57, 71, 73], Goldberg Anxiety and Depression Scale [19, 46, 47], Philadelphia Geriatric Morale Scale [32, 35, 74], Cornell Scale for Depression in Dementia [29, 52, 53], Beck Depression Inventory [42, 70], Yesavage Scale [65], and the Profile of Mood State Short Form [77].

### Effect of intervention

Across the 63 included studies, intention to treat analyses were performed in 21 studies. The included studies reporting significant improvements in physical function, QOL and/or mental health in the intervention group compared with the comparator group/s are summarised in Table 2.

**Table 2:** Summary of included studies reporting significant improvements in outcomes in the intervention compared with comparator group/s

Outcome (n included studies measuring this outcome)	Intervention vs usual care n (%)^a^	Intervention vs other physical activity intervention/s n (%)^a^
Physical function (*n* = 55)		
Mobility	19 (35%)	5 (9%)
Balance	10 (18%)	5 (9%)
SPPB	4 (7%)	2 (4%)
Physical activity	3 (5%)	
Frailty	2 (4%)	
ADLs	10 (18%)	3 (5%)
Quality of Life (*n* = 20)	6 (30%)	2 (10%)
Mental health (*n* = 19)		
Depression	4 (21%)	1 (5%)

^a^n studies reporting significant improvement in the outcome of interest compared to comparator group/s (% of studies measuring that outcome that reported significant improvement)

#### Physical function

Of the 55 included studies which measured physical function, 33 (60%) reported significant improvement in physical function measures in the intervention group compared with the comparator group/s. Of these, 25 studies compared a physical activity intervention to either usual care or a social/recreational activity and found improvements in relation to physical function for mobility (*n* = 19) [18, 20, 27, 29, 30, 36, 37, 40, 41, 43, 44, 56, 63–65, 67, 68, 70, 75], balance (*n* = 10) [20, 36, 40, 44, 52–54, 68, 70, 75], ADLs (*n* = 10) [18, 27, 29, 40, 53, 60, 63, 65, 68, 76], the SPPB (*n* = 4) [20, 21, 56, 67], physical activity (*n* = 3) [36, 64, 67], and frailty (*n* = 2) [65, 67]. Eight studies that compared two or more physical activity interventions reported a significant improvement in physical functioning for the primary intervention group in mobility (*n* = 5) [25, 34, 46, 47, 79], balance (*n* = 5) [25, 28, 47, 48, 79], ADLs (*n* = 3) [23, 48, 79] and the SPPB (*n* = 2) [47, 48].

#### Quality of life

Eight of the 20 (40%) included studies measuring QOL reported significant improvements in the intervention group compared with the comparator group/s for this outcome. Six of these studies compared a physical activity intervention versus usual care / social activity [18, 31, 42, 64, 70, 71], and two studies compared two or more physical activity interventions [45, 48].

#### Mental health

Of the 19 included studies measuring outcomes relating to mental health, five (26%) reported significant benefits for mental health in the intervention compared with comparator group/s, with all of these studies measuring depression. Of these, four studies [42, 57, 70, 71] compared a physical activity intervention with usual care, and one study [48] compared multiple physical activity interventions.

### Features of reablement strategies

Table 3 shows the specific components of the reablement strategies identified in the included studies with effectiveness in improving physical function measures (*n* = 33). Multi-component interventions including two or more components (aerobic, balance, strength, functional exercise) were the most common among studies to demonstrate effectiveness in relation to mobility (*n* = 11), balance (*n* = 11), ADLs (*n* = 5) and the SPPB (*n* = 3). Interventions including technology were the most common type for increasing physical activity (*n* = 2), and there was only one multi-component and one technology intervention demonstrating effectiveness for the frailty outcome measure. The features of reablement strategies which did not report preliminary effectiveness for physical function are also reported in Table 3.

**Table 3:** Features of reablement strategies that have demonstrated preliminary effectiveness in addressing physical function in older adults in RAC

Intervention type	Outcomes					
	Mobility	Balance	ADLs	SPPB	Physical activity	Frailty
Multi-component	[20, 25, 27, 34, 36, 40, 41, 46, 47, 65, 68]	[20, 25, 28, 34, 36, 40, 47, 52–54, 68]	[27, 40, 53, 65, 68]	[20, 21, 47]	[36]	[65]
	**[**24**,**^32^**,**^35^**,**^52^**,**^53^**,**^74^**]**	**[**27**,**^35^**]**	**[**24**,**^35^**,**^41^**,**^52^**,**^54^**,**^74^**,**^80^**]**	**[**24**,**^46^**]**	**[**47**]**	**[**46**]**
Strength	[30, 56]		[23, 48, 60]	[48, 56]		
	**[**38**,**^39^**,**^49^**]**		**[**73**]**			
Aerobic	[29, 44, 63, 75]	[44, 75]	[29, 63]			
	**[**58**,**^59^**,**^66^**]**	**[**66**]**	**[**44**,**^59^**]**			
Tai Chi	**[**33**]**	**[**78**]**	**[**33**]**			
Dance	[37, 43]					
Whole body vibration	[18, 79]		[18, 79]			
	**[**22**,**^26^**,**^50^**,**^51^**,**^72^**]**	**[**22**,**^26^**,**^50^**,**^51^**,**^72^**]**		**[**50**]**		
Hand ball training			[76]			
Technology	[64, 67, 70]	[70]		[67]	[64, 67]	[67]
	**[**62**,**^69^**]**	**[**62**,**^69^**]**	**[**69**]**			

Bolded text represents studies which measured the outcome but did not report a significant between group difference

*ADLs* Activities of daily living, *SPPB* Short physical performance battery

### Feasibility

#### Compliance

Across all included studies, 59% (*n* = 37) reported participant compliance with the intervention. Of these studies, 35% (*n* = 13) had compliance rates greater than 90% [19–22, 26, 27, 40, 46, 47, 62, 63, 75, 79], 57% (*n* = 21) between 70 and 90% [23–25, 29, 32, 37, 39, 49–54, 57, 60, 64, 66, 67, 71, 72, 80], and 8% (*n* = 3) below 70% [31, 33, 65].

#### Acceptability / satisfaction

Four studies [24, 67, 69, 79] reported on the acceptability of the physical activity intervention/s. Three of these studies collected qualitative feedback from participants [24, 69, 79] with participants reporting mainly positive feedback (e.g. that the exercises were fun and enjoyable). One study collected information on acceptability in terms of difficulty of the exercises and enjoyment [67]. The majority of participants in this study reported that most of the exercises were easy (70%) and highly enjoyed (63%).

#### Adverse events

In total, 32 (51%) of included studies provided information in relation to the occurrence of adverse events. The majority of these studies (*n* = 24, 75%) reported no adverse events. Adverse events were reported in several studies (*n* = 8), in particular, intervention participants experienced pain / soreness [22, 24, 38, 40, 49, 51, 80], fatigue [38, 40] and electrocardiographic changes [80]. One study also reported that two participants experienced a fall during the intervention [67].

#### Cost effectiveness

None of the included studies reported cost effectiveness relating to the interventions.

## Discussion

This review aimed to scope the evidence for reablement strategies for older adults in RAC. Specifically, the review sought to explore the effectiveness of interventions relating to physical function, QOL and mental health, the features of effective interventions, and feasibility including compliance, acceptability, adverse events and cost effectiveness. There were many studies published fulfilling the eligibility criteria for the review in the last 10 years, with an apparent surge in research in this setting, particularly in Europe, however, only relatively few were identified in the United States, Asia and Oceania. There was wide variability in the types and features of reablement interventions in the included studies, and the outcome measures that were used to measure physical function. Over half of the studies that measured physical function reported on the effectiveness of the intervention, 40% of studies that measured QOL were shown to be effective for this measure and just over a quarter of the studies including mental health were effective. Over half of the studies measured compliance with the intervention and adverse events, while only 6 % of included studies measured participant satisfaction or acceptability. None of the included studies measured or reported cost effectiveness of the intervention.

There was variability between the included studies in the numbers of participants, however, on average the participants in the studies were representative of the types of residents in RAC in high-income countries. The gender balance (70% female) and average age (83 years) across the included studies is comparable [81]. The studies all included residents of RAC, but there was also representation of subpopulations such as people with dementia, Alzheimer’s disease, and osteopenia. Despite the use of an a priori definition of RAC in this review, it was at times difficult to establish whether the settings described in the literature fulfilled these criteria. Terms found in the literature included nursing home, residential care home, residential care facility, long-term care home, long-term care facility, assisted living facility and institution. There is a need for clear definition of terms for this setting in the literature to determine the relevance and generalisability of published findings.

Of the interventions detailed across the included studies, it was interesting that none were explicitly termed ‘reablement’ or ‘restorative’ care programs. A recent scoping review of the literature on the integration of physical activity in reablement for community dwelling adults also included interventions that explored the concept of reablement by being person-centred and aiming to improve functional ability [82]. In contrast, the majority of included studies in this previous review used the terms reablement or restorative care, with only a few included studies including other descriptions. The lack of ‘reablement’ terminology found in the current review may reflect the recency in which this term has been used, both in recommendations pertaining to aged care, and in the scientific literature in the RAC setting. Reablement is similar to the approach of function-focused care (term mainly used in the United States), while the concept of reablement has been predominantly used in the United Kingdom, Australia and New Zealand [83]. There has been considerable debate surrounding the definition and true meaning of reablement, with an internationally accepted definition published in the last 12 months [83]. Despite the challenges regarding terminology, we are confident that the interventions within the included studies fit the description of reablement programs, in that they were targeted, goal-oriented programs aimed at maintaining or improving physical function in this setting. The focus of this review was on exploring the effectiveness, features of strategies and feasibility. While information was collected regarding participant adherence and satisfaction with the intervention, resident goals, barriers, facilitators, or other perceptions of intervention implementation were not extracted. This is an important area for future research.

There was variability in the exercise prescription in terms of the overall duration of the intervention, frequency, and components of exercise. Most commonly, interventions lasted for between 3 and 4 months, with sessions conducted two to three times per week. This is consistent with previous literature which describes the time required to form habitual health behaviours such as physical activity [84]. However, the current global guidelines [85] recommend that older adults (≥ 65 years) should accumulate at least 150 min of moderate intensity physical activity throughout the week. These guidelines also recommend that, at least three times per week, older adults should engage in varied multicomponent physical activity that emphasises functional balance and strength training, to prevent falls and enhance functional capacity. In line with these recommendations, the most common type of intervention in the included studies was multi-component, with interventions including two or more components of activity such as aerobic, strength and balance exercise. There is a growing body of evidence to support the potential for interactive gaming technologies in RAC to improve mood, engagement and wellbeing [86–88]. The use of these technologies to engage and support the delivery of interventions is an area of potential growth for intervention development and implementation in RAC.

Regarding the people associated with delivery of the interventions in the included studies, it was noted that physiotherapists were the most common, while exercise specialists such as exercise scientists or physiologists were reported in comparably few studies. There is a growing trend for interprofessional delivery of programs in the aged care setting [89], however, this may not be representative within research studies. While 38% of the included studies reported information relating to intervention development, only four studies reported considering the views and needs of the residents in the development and implementation of the intervention. None of the included studies reported wider stakeholder consultation. Given the many barriers identified in the previous literature regarding sustainability of reablement approaches in aged care [10], and the identified need to explore feasibility of interventions [14], stakeholders including the residents themselves should be consulted and involved in the development and implementation of such strategies [90].

There were many different outcome measures used to measure physical function across the included studies, making pooling of results and comparison between studies difficult. There is a clear need for a set of core outcomes for physical function to be established for both research and clinical practice in this setting. While the majority of the included studies included some measure of physical function, just under a third measured QOL or mental health. This is surprising given the importance of these outcomes to the overall health and wellbeing of older adults, and the associations reported in the literature between activity levels and physical / mental wellbeing [91].

Despite the diversity in intervention types and implementation, over half of the studies measuring physical function demonstrated effectiveness, while 40% of the studies measuring QOL reported effectiveness and about a quarter of those measuring mental health outcomes were effective. These findings demonstrate that while we have evidence that reablement programs are effective in improving physical function, additional work is needed to investigate sustainability and scalability of approaches, and potential impact on QOL and mental health of residents. Finally, while over half of the included studies measured compliance with the intervention and adverse events, it was surprising that few studies measured resident satisfaction or acceptability. The focus on intervention effectiveness rather than the views and preferences of the residents is concerning, and a potential barrier for longer term impact and scalability of these interventions. The lack of studies investigating cost effectiveness in the current review may reflect a lack of priority, knowledge or skills within research teams in health economics. Previous reviews have identified a lack of studies of cost-effectiveness in the RAC context, in contrast to the relative abundance of these studies in the healthcare sector [92, 93]. Given the growing proportion of the population using aged care services, it is crucial that potentially cost-effective models of aged care are identified for implementation. These factors must be taken into consideration in the design and implementation of strategies to address physical deconditioning in RAC to ensure scalability of programs.

This scoping review of the literature has several methodological strengths. The search strategy was peer-reviewed, and every effort was made to include all possible terms for older adults, RAC, reablement and the relevant outcomes of interest, ensuring breadth and rigour. However, given the variability in terminology identified in this review, it is possible that some potentially relevant terms were missed. We prospectively registered the review protocol, and followed the PRISMA guidelines for scoping reviews [15] as well as scoping review methodological recommendations [16, 17]. There are also some limitations. Given the variability in intervention types, delivery, and outcome measures, it is difficult to make conclusions regarding the effectiveness of interventions for physical function, QOL and mental health in this setting.

## Conclusions

This review scoped the current context of the evidence for reablement interventions for older adults in RAC. While there is a body of literature reporting effectiveness of interventions in relation to improvements in physical function, there remains a paucity of literature investigating QOL and mental health, as well as investigating compliance, acceptability and cost effectiveness. Areas for future research include establishment of a core outcome set for physical function of older adults in RAC for both researchers and clinical practice, technology-based interventions, and exploration of co-design and implementation involving wide stakeholder consultation for intervention in this setting. There is also a clear need for work in this area in the Australian context.

## Supplementary Information

**Additional file 1.** Detailed search strategy.

**Additional file 2.** Summary of included studies.

## Data Availability

All data generated or analysed during this study are included in this published article [and its supplementary information files].

## Abbreviations

ADL: Activities of daily living
FIM: Functional Independence Measure
QOL: Quality of life
RAC: Residential aged care
RCT: Randomised controlled trial
SPPB: Short Physical Performance Battery
TUG: Timed Up and Go
6MWT: 6 Minute Walk Test

## References

[1] Australian Institute of Health and Welfare (2019). Aged care.

[2] Reid N, Keogh JW, Swinton P, Gardiner PA, Henwood TR (2018). The association of sitting time with sarcopenia status and physical performance at baseline and 18-month follow-up in the residential aged care setting. J Aging Phys Act.

[3] Theou O, Tan EC, Bell JS, Emery T, Robson L, Morley JE (2016). Frailty levels in residential aged care facilities measured using the frailty index and FRAIL-NH scale. J Am Geriatr Soc.

[4] Senior HE, Henwood TR, Beller EM, Mitchell GK, Keogh JW (2015). Prevalence and risk factors of sarcopenia among adults living in nursing homes. Maturitas.

[5] Dent E, Lien C, Lim WS, Wong WC, Wong CH, Ng TP (2017). The Asia-Pacific clinical practice guidelines for the management of frailty. J Am Med Dir Assoc.

[6] Morley JE, Vellas B, Van Kan GA, Anker SD, Bauer JM, Bernabei R (2013). Frailty consensus: a call to action. J Am Med Dir Assoc.

[7] Taylor D, Barrie H, Lange J, Thompson M, Theou O, Visvanathan R (2019). Geospatial modelling of the prevalence and changing distribution of frailty in Australia–2011 to 2027. Exp Gerontol.

[8] Fien S, Henwood T, Climstein M, Rathbone E, Keogh JW (2019). Exploring the feasibility, sustainability and the benefits of the GrACE+ GAIT exercise programme in the residential aged care setting. PeerJ.

[9] Hewitt J, Goodall S, Clemson L, Henwood T, Refshauge K (2018). Progressive resistance and balance training for falls prevention in long-term residential aged care: a cluster randomized trial of the sunbeam program. J Am Med Dir Assoc.

[10] Benjamin K, Edwards N, Ploeg J, Legault F (2014). Barriers to physical activity and restorative care for residents in long-term care: a review of the literature. J Aging Phys Act.

[11] Dyer S, van den Berg M, Barnett K, Brown A, Johnstone G, Laver K (2019). Review of innovative models of aged care: report prepared for the Royal Commission into Aged Care Quality and Safety.

[12] Aspinal F, Glasby J, Rostgaard T, Tuntland H, Westendorp RG (2016). New horizons: Reablement-supporting older people towards independence. Age Ageing.

[13] Australian Association of Gerontology (2019). Fact Sheet 3: Australian approaches to reablement in residential aged care.

[14] Crocker T, Forster A, Young J, Brown L, Ozer S, Smith J, et al. Physical rehabilitation for older people in long-term care. Cochrane Database Syst Rev. 2013;(2). 10.1002/14651858.CD004294.pub3. 10.1002/14651858.CD004294.pub3 PMC11930398 23450551

[15] Tricco AC, Lillie E, Zarin W, O'Brien KK, Colquhoun H, Levac D (2018). PRISMA extension for scoping reviews (PRISMA-ScR): checklist and explanation. Ann Intern Med.

[16] Arksey H, O'Malley L (2005). Scoping studies: towards a methodological framework. Int J Soc Res Methodol.

[17] Levac D, Colquhoun H, O'Brien KK (2010). Scoping studies: advancing the methodology. Implement Sci.

[18] Álvarez-Barbosa F, del Pozo-Cruz J, del Pozo-Cruz B, Alfonso-Rosa RM, Rogers ME, Zhang Y (2014). Effects of supervised whole body vibration exercise on fall risk factors, functional dependence and health-related quality of life in nursing home residents aged 80+. Maturitas.

[19] Arrieta H, Rezola-Pardo C, Kortajarena M, Hervás G, Gil J, Yanguas JJ (2020). The impact of physical exercise on cognitive and affective functions and serum levels of brain-derived neurotrophic factor in nursing home residents: a randomized controlled trial. Maturitas.

[20] Arrieta H, Rezola-Pardo C, Zarrazquin I, Echeverria I, Yanguas JJ, Iturburu M (2018). A multicomponent exercise program improves physical function in long-term nursing home residents: a randomized controlled trial. Exp Gerontol.

[21] Arrieta H, Rezola-Pardo C, Gil SM, Virgala J, Iturburu M, Antón I (2019). Effects of multicomponent exercise on frailty in long-term nursing homes: a randomized controlled trial. J Am Geriatr Soc.

[22] Beaudart C, Maquet D, Mannarino M, Buckinx F, Demonceau M, Crielaard J-M (2013). Effects of 3 months of short sessions of controlled whole body vibrations on the risk of falls among nursing home residents. BMC Geriatr.

[23] Benavent-Caballer V, Rosado-Calatayud P, Segura-Ortí E, Amer-Cuenca J, Lisón J (2014). Effects of three different low-intensity exercise interventions on physical performance, muscle CSA and activities of daily living: a randomized controlled trial. Exp Gerontol.

[24] Bischoff LL, Cordes T, Meixner C, Schoene D, Voelcker-Rehage C, Wollesen B. Can cognitive-motor training improve physical functioning and psychosocial wellbeing in nursing home residents? A randomized controlled feasibility study as part of the PROCARE project. Aging Clin Exp Res. 2021;33(4):943–56. 10.1007/s40520-020-01615-y 32537707

[25] Bossers WJ, van der Woude LH, Boersma F, Hortobágyi T, Scherder EJ, van Heuvelen MJ (2015). A 9-week aerobic and strength training program improves cognitive and motor function in patients with dementia: a randomized, controlled trial. Am J Geriatr Psychiatry.

[26] Buckinx F, Beaudart C, Maquet D, Demonceau M, Crielaard J-M, Reginster J-Y (2014). Evaluation of the impact of 6-month training by whole body vibration on the risk of falls among nursing home residents, observed over a 12-month period: a single blind, randomized controlled trial. Aging Clin Exp Res.

[27] Cadore EL, Casas-Herrero A, Zambom-Ferraresi F, Idoate F, Millor N, Gómez M (2014). Multicomponent exercises including muscle power training enhance muscle mass, power output, and functional outcomes in institutionalized frail nonagenarians. Age.

[28] Cakar E, Dincer U, Kiralp M, Cakar D, Durmus O, Kilac H (2010). Jumping combined exercise programs reduce fall risk and improve balance and life quality of elderly people who live in a long-term care facility. Eur J Phys Rehabil Med.

[29] Cancela JM, Ayán C, Varela S, Seijo M (2016). Effects of a long-term aerobic exercise intervention on institutionalized patients with dementia. J Sci Med Sport.

[30] Carral JMC, Rodríguez AL, Cardalda IM, Bezerra JPAG (2019). Muscle strength training program in nonagenarians–a randomized controlled trial. Rev Assoc Méd Bras.

[31] Cichocki M, Quehenberger V, Zeiler M, Adamcik T, Manousek M, Stamm T (2015). Effectiveness of a low-threshold physical activity intervention in residential aged care–results of a randomized controlled trial. Clin Interv Aging.

[32] Conradsson M, Littbrand H, Lindelöf N, Gustafson Y, Rosendahl E (2010). Effects of a high-intensity functional exercise programme on depressive symptoms and psychological well-being among older people living in residential care facilities: a cluster-randomized controlled trial. Aging Ment Health.

[33] Dechamps A, Diolez P, Thiaudière E, Tulon A, Onifade C, Vuong T (2010). Effects of exercise programs to prevent decline in health-related quality of life in highly deconditioned institutionalized elderly persons: a randomized controlled trial. Arch Intern Med.

[34] Espejo-Antúnez L, Pérez-Mármol JM, de los Ángeles Cardero-Durán M, Toledo-Marhuenda JV, Albornoz-Cabello M (2020). The effect of proprioceptive exercises on balance and physical function in institutionalized older adults: a randomized controlled trial. Arch Phys Med Rehabil.

[35] Frändin K, Grönstedt H, Helbostad JL, Bergland A, Andresen M, Puggaard L (2016). Long-term effects of individually tailored physical training and activity on physical function, well-being and cognition in scandinavian nursing home residents: a randomized controlled trial. Gerontology.

[36] Grönstedt H, Frändin K, Bergland A, Helbostad JL, Granbo R, Puggaard L (2013). Effects of individually tailored physical and daily activities in nursing home residents on activities of daily living, physical performance and physical activity level: a randomized controlled trial. Gerontology.

[37] Holmerová I, MacHácová K, Vanková H, Veleta P, Jurasková B, Hrnciariková D (2010). Effect of the exercise dance for seniors (EXDASE) program on lower-body functioning among institutionalized older adults. J Aging Health.

[38] i Iranzo MÀC, Balasch-Bernat M, Tortosa-Chuliá MÁ, Balasch-Parisi S (2018). Effects of resistance training of peripheral muscles versus respiratory muscles in older adults with sarcopenia who are institutionalized: a randomized controlled trial. J Aging Phys Act.

[39] Johnen B, Schott N (2018). Feasibility of a machine vs free weight strength training program and its effects on physical performance in nursing home residents: a pilot study. Aging Clin Exp Res.

[40] Kocic M, Stojanovic Z, Nikolic D, Lazovic M, Grbic R, Dimitrijevic L (2018). The effectiveness of group Otago exercise program on physical function in nursing home residents older than 65 years: a randomized controlled trial. Arch Gerontol Geriatr.

[41] Kovacs E, Sztruhar Jonasne I, Karoczi C, Korpos A, Gondos T (2013). Effects of a multimodal exercise program on balance, functional mobility and fall risk in older adults with cognitive impairment: a randomized controlled single-blind study. Eur J Phys Rehabil Med.

[42] Lok N, Lok S, Canbaz M (2017). The effect of physical activity on depressive symptoms and quality of life among elderly nursing home residents: randomized controlled trial. Arch Gerontol Geriatr.

[43] Machacova K, Vankova H, Volicer L, Veleta P, Holmerova I (2017). Dance as prevention of late life functional decline among nursing home residents. J Appl Gerontol.

[44] Naczk M, Marszalek S, Naczk A (2020). Inertial training improves strength, balance, and gait speed in elderly nursing home residents. Clin Interv Aging.

[45] Nawrat-Szoltysik A, Miodonska Z, Opara J, Polak A, Matyja B, Malecki A (2019). Effect of physical activity on the quality of life in osteoporotic females living in residential facilities: a randomized controlled trial. J Geriatr Phys Ther.

[46] Rezola-Pardo C, Arrieta H, Gil SM, Zarrazquin I, Yanguas JJ, López MA (2019). Comparison between multicomponent and simultaneous dual-task exercise interventions in long-term nursing home residents: the Ageing-ONDUAL-TASK randomized controlled study. Age Ageing.

[47] Rezola-Pardo C, Rodriguez-Larrad A, Gomez-Diaz J, Lozano-Real G, Mugica-Errazquin I, Patiño MJ (2020). Comparison between multicomponent exercise and walking interventions in long-term nursing homes: a randomized controlled trial. The Gerontologist.

[48] Sahin UK, Kirdi N, Bozoglu E, Meric A, Buyukturan G, Ozturk A (2018). Effect of low-intensity versus high-intensity resistance training on the functioning of the institutionalized frail elderly. Int J Rehabil Res.

[49] Serra-Rexach JA, Bustamante-Ara N, Hierro Villarán M, González Gil P, Sanz Ibanez MJ, Blanco Sanz N (2011). Short-term, light-to moderate-intensity exercise training improves leg muscle strength in the oldest old: a randomized controlled trial. J Am Geriatr Soc.

[50] Sievänen H, Karinkanta S, Moisio-Vilenius P, Ripsaluoma J (2014). Feasibility of whole-body vibration training in nursing home residents with low physical function: a pilot study. Aging Clin Exp Res.

[51] Sitjà-Rabert M, Martínez-Zapata MJ, Vanmeerhaeghe AF, Abella FR, Romero-Rodríguez D, Bonfill X (2015). Effects of a whole body vibration (WBV) exercise intervention for institutionalized older people: a randomized, multicentre, parallel, clinical trial. J Am Med Dir Assoc.

[52] Telenius EW, Engedal K, Bergland A (2015). Long-term effects of a 12 weeks high-intensity functional exercise program on physical function and mental health in nursing home residents with dementia: a single blinded randomized controlled trial. BMC Geriatr.

[53] Telenius EW, Engedal K, Bergland A (2015). Effect of a high-intensity exercise program on physical function and mental health in nursing home residents with dementia: an assessor blinded randomized controlled trial. PLoS One.

[54] Toots A, Littbrand H, Lindelöf N, Wiklund R, Holmberg H, Nordström P (2016). Effects of a high-intensity functional exercise program on dependence in activities of daily living and balance in older adults with dementia. J Am Geriatr Soc.

[55] Tuunainen E, Rasku J, Jäntti P, Moisio-Vilenius P, Mäkinen E, Toppila E (2013). Postural stability and quality of life after guided and self-training among older adults residing in an institutional setting. Clin Interv Aging.

[56] Urzi F, Marusic U, Ličen S, Buzan E (2019). Effects of elastic resistance training on functional performance and Myokines in older women—a randomized controlled trial. J Am Med Dir Assoc.

[57] Vankova H, Holmerova I, Machacova K, Volicer L, Veleta P, Celko AM (2014). The effect of dance on depressive symptoms in nursing home residents. J Am Med Dir Assoc.

[58] Varela S, Ayán C, Cancela JM, Martín V (2012). Effects of two different intensities of aerobic exercise on elderly people with mild cognitive impairment: a randomized pilot study. Clin Rehabil.

[59] Varela S, Cancela JM, Seijo-Martinez M, Ayán C (2018). Self-paced cycling improves cognition on institutionalized older adults without known cognitive impairment: a 15-month randomized controlled trial. J Aging Phys Act.

[60] Venturelli M, Lanza M, Muti E, Schena F (2010). Positive effects of physical training in activity of daily living–dependent older adults. Exp Aging Res.

[61] Wiśniowska-Szurlej A, Ćwirlej-Sozańska A, Wołoszyn N, Sozański B, Wilmowska-Pietruszyńska A (2020). Effects of physical exercises and verbal stimulation on the functional efficiency and use of free time in an older population under institutional care: a randomized controlled trial. J Clin Med.

[62] Yeşilyaprak SS, Yıldırım MŞ, Tomruk M, Ertekin Ö, Algun ZC (2016). Comparison of the effects of virtual reality-based balance exercises and conventional exercises on balance and fall risk in older adults living in nursing homes in Turkey. Physiother Theory Pract.

[63] Venturelli M, Scarsini R, Schena F (2011). Six-month walking program changes cognitive and ADL performance in patients with Alzheimer. Am J Alzheimers Dis Other Dement.

[64] Dillon K, Prapavessis H (2020). REducing SEDENTary behavior among mild to moderate cognitively impaired assisted living residents: a pilot randomized controlled trial (RESEDENT study). J Aging Phys Act.

[65] Ferreira CB, Teixeira PS, Alves dos Santos G, Dantas Maya AT, Souza VC, Americano do Brasil P (2018). Effects of a 12-week exercise training program on physical function in institutionalized frail elderly. J Aging Res.

[66] Harris JB, Johnson CS (2017). The impact of physical versus social activity on the physical and cognitive functioning of seniors with dementia. Act Adapt Aging.

[67] Lauzé M, Martel DD, Aubertin-Leheudre M (2017). Feasibility and effects of a physical activity program using gerontechnology in assisted living communities for older adults. J Am Med Dir Assoc.

[68] Moreira NB, Gonçalves G, da Silva T, Zanardini FEH, Bento PCB (2018). Multisensory exercise programme improves cognition and functionality in institutionalized older adults: a randomized control trial. Physiother Res Int.

[69] Padala KP, Padala PR, Malloy TR, Geske JA, Dubbert PM, Dennis RA (2012). Wii-fit for improving gait and balance in an assisted living facility: a pilot study. J Aging Res.

[70] Rica RL, Shimojo GL, Gomes MC, Alonso AC, Pitta RM, Santa-Rosa FA (2020). Effects of a Kinect-based physical training program on body composition, functional fitness and depression in institutionalized older adults. Geriatr Gerontol Int.

[71] Hsu C-Y, Moyle W, Cooke M, Jones C (2016). Seated Tai Chi versus usual activities in older people using wheelchairs: a randomized controlled trial. Complement Ther Med.

[72] Lam FM, Chan PF, Liao L, Woo J, Hui E, Lai CW (2018). Effects of whole-body vibration on balance and mobility in institutionalized older adults: a randomized controlled trial. Clin Rehabil.

[73] Liu I-T, Lee W-J, Lin S-Y, Chang S-T, Kao C-L, Cheng Y-Y (2020). Therapeutic effects of exercise training on elderly patients with dementia: a randomized controlled trial. Arch Phys Med Rehabil.

[74] Takeuchi R, Hatano Y, Yamasaki M (2011). The influence of different exercise intervention programs on changes in quality of life and activity of daily living levels among geriatric nursing home residents. J Phys Ther Sci.

[75] Tsaih P-L, Shih Y-L, Hu M-H (2012). Low-intensity task-oriented exercise for ambulation-challenged residents in long-term care facilities: a randomized, controlled trial. Am J Phys Med Rehabil.

[76] Wei X-h, Ji L-l (2014). Effect of handball training on cognitive ability in elderly with mild cognitive impairment. Neurosci Lett.

[77] Hsu C-Y, Moyle W, Cooke M, Jones C (2016). Seated T’ai Chi in older Taiwanese people using wheelchairs: a randomised controlled trial investigating mood states and self-efficacy. J Altern Complement Med.

[78] Saravanakumar P, Johanna Higgins I, Jane van der Riet P, Marquez J, Sibbritt D (2014). The influence of tai chi and yoga on balance and falls in a residential care setting: a randomised controlled trial. Contemp Nurse.

[79] Wadsworth D, Lark S (2020). Effects of whole-body vibration training on the physical function of the frail elderly: an open, randomized controlled trial. Arch Phys Med Rehabil.

[80] Lorenz RA, Gooneratne N, Cole CS, Kleban MH, Kalra GK, Richards KC (2012). Exercise and social activity improve everyday function in long-term care residents. Am J Geriatr Psychiatry.

[81] Australian Institute of Health and Welfare (2018). Older Australia at a glance.

[82] Mjøsund HL, Moe CF, Burton E, Uhrenfeldt L (2020). Integration of physical activity in reablement for community dwelling older adults: a systematic scoping review. J Multidiscip Healthc.

[83] Metzelthin SF, Rostgaard T, Parsons M, Burton E. Development of an internationally accepted definition of reablement: a Delphi study. Ageing Soc. 2020:1–16. https://www.cambridge.org/core/journals/ageing-and-society/article/development-of-an-internationally-accepted-definition-of-reablement-a-delphi-study/CE189681CD52A59259F4331543A71A23.

[84] Aarts H, Paulussen T, Schaalma H (1997). Physical exercise habit: on the conceptualization and formation of habitual health behaviours. Health Educ Res.

[85] World Health Organization (2020). WHO guidelines on physical activity and sedentary behaviour: at a glance.

[86] Gunst M, De Meyere I, Willems H, Schoenmakers B. Effect of exergaming on wellbeing of residents in a nursing home: a single blinded intervention study. Aging Clin Exp Res. 2021. 10.1007/s40520-021-01903-1. 10.1007/s40520-021-01903-1 PMC8794998 34156650

[87] Keogh JW, Power N, Wooller L, Lucas P, Whatman C (2014). Physical and psychosocial function in residential aged-care elders: effect of Nintendo Wii Sports games. J Aging Phys Act.

[88] Baker S, Waycott J, Robertson E, Carrasco R, Neves BB, Hampson R (2020). Evaluating the use of interactive virtual reality technology with older adults living in residential aged care. Inf Process Manag.

[89] Tsakitzidis G, Timmermans O, Callewaert N, Verhoeven V, Lopez-Hartmann M, Truijen S (2016). Outcome indicators on interprofessional collaboration interventions for elderly. Int J Integr Care.

[90] Harvey G, Kitson A. Implementing evidence-based practice in healthcare: a facilitation guide. London and New York: Routledge; 2015.

[91] Edvardsson D, Petersson L, Sjogren K, Lindkvist M, Sandman PO (2014). Everyday activities for people with dementia in residential aged care: associations with person-centredness and quality of life. Int J Older People Nursing.

[92] Easton T, Milte R, Crotty M, Ratcliffe J (2016). Advancing aged care: a systematic review of economic evaluations of workforce structures and care processes in a residential care setting. Cost Eff Resour Alloc.

[93] Easton T, Milte R, Crotty M, Ratcliffe J (2017). Where’s the evidence? A systematic review of economic analyses of residential aged care infrastructure. BMC Health Serv Res.

