# Supplementary material for: Re-thinking reablement strategies for older adults in residential aged care: a scoping review
Source: BMC Geriatr. 2021 Nov 30;21:667. doi: 10.1186/s12877-021-02627-7 (PMC8638477; doi:10.1186/s12877-021-02627-7)
Supplement: Supplementary file 1 — Additional file 1. Detailed search strategy. [file 12877_2021_2627_MOESM1_ESM.docx]

**ADDITIONAL FILE 1: DETAILED SEARCH STRATEGY**

MEDLINE, EMBASE, CINAHL, Cochrane Central Register of Controlled Trials, Cochrane Database of Systematic Reviews were searched using both subject heading and keyword searches where possible.

**Databases: MEDLINE and EMBASE**

1. (elder* or geriatric* or old* adult* or old* person or old* people or old age or senior* or aged or ageing or aging or late* life).ti,ab.

2. geriatrics/ or aged/ or aging/ aged, 80 and over/

3. 1 or 2

4. (residential care or aged care or nursing home* or residential facilit* or assisted living or care home* or old age home* or long-term care or institutionali* or extended care facilit* or home* for the aged).ti,ab.

5. homes for the aged/ or residential facilities/ or assisted living facilities/

6. 4 or 5

7. (restorative care or reablement or re-ablement or rehabilitat* or reactivation program or re-activation program or exercise or strength train* or resistance train* or physical activity).ti,ab.

8. exercise therapy/ rehabilitation/

9. 7 or 8

10. (function or independence or mobility or walking or quality of life or QOL or HrQOL or activit* of daily living or ADL or mood or depression or anxiety or mental health or well-being or wellbeing).ti,ab.

11. activities of daily living/ or quality of life/

12. 10 or 11

13. 3 and 6 and 9 and 12

**Database: CINAHL**

1. TI (elder* or geriatric* or “old* adult*” or “old* person” or “old* people” or “old age” or senior* or aged or ageing or aging or “late* life”) OR AB (elder* or geriatric* or “old* adult*” or “old* person” or “old* people” or “old age” or senior* or aged or ageing or aging or “late* life”)

2. (MH Geriatrics) OR (MH Aged) OR (MH Aging) OR (MH Aged, 80 and over)

3. 1 or 2

4. TI (“residential care” or “aged care” or “nursing home*” or “residential facilit*” or “assisted living” or “care home*” or “old age home*” or “long-term care” or institutionali* or “extended care facilit*” or “home* for the aged”) OR AB (“residential care” or “aged care” or “nursing home*” or “residential facilit*” or “assisted living” or “care home*” or “old age home*” or “long-term care” or institutionali* or “extended care facilit*” or “home* for the aged”)

5. (MH Residential Facilities) OR (MH Assisted Living)

6. 4 or 5

7. TI (“restorative care” or reablement or re-ablement or rehabilitat* or “reactivation program” or “re-activation program” or exercise or “strength train*” or “resistance train*” or “physical activity”) OR AB (“restorative care” or reabalement or re-ablement or rehabilitat* or “reactivation program” or “re-activation program” or exercise or “strength train*” or “resistance train*” or “physical activity”)

8. (MH Therapeutic Exercise) OR (MH Rehabilitation)

9. 7 or 8

10. TI (function or independence or mobility or walking or “quality of life” or QOL or HrQOL or “activit* daily living” or ADL or mood or depression or anxiety or “mental health” or well-being or wellbeing) OR AB (function or independence or mobility or walking or “quality of life” or QOL or HrQOL or “activit* daily living” or ADL or mood or depression or anxiety or “mental health” or well-being or wellbeing)

11. (MH Activities of Daily Living) OR (MH Quality of Life)

12. 10 or 11

13. 3 and 6 and 9 and 12

**Databases: Cochrane Central Register of Controlled Trials and Cochrane Database of Systematic Reviews**

1. (elder* or geriatric* or old* adult* or old* person or old* people or old age or senior* or aged or ageing or aging or late* life):ti,ab,kw

2. MeSH descriptor: [Geriatrics] this term only

3. MeSH descriptor: [Aged] this term only

4. MeSH descriptor: [Aged, 80 and over] this term only

5. #1 or #2 or #3 or #4

6. (residential care or aged care or nursing home* or residential facilit* or assisted living or care home* or old age home* or long-term care or institutionali* or extended care facilit* or home* for the aged):ti,ab,kw

7. MeSH descriptor: [Homes for the aged] this term only

8. MeSH descriptor: [Residential Facilities] this term only

9. MeSH descriptor: [Assisted Living Facilities] this term only

10. #6 or #7 or #8 or #9

11. (restorative care or reablement or re-ablement or rehabilitat* or reactivation program or re-activation program or exercise or strength train* or resistance train* or physical activity):ti,ab,kw

12. MeSH descriptor: [Exercise Therapy] this term only

13. MeSH descriptor: [Rehabilitation] this term only

14. #11 or #12 or #13

15. (function or independence or mobility or walking or quality of life or QOL or HrQOL or activit* daily living or ADL or mood or depression or anxiety or mental health or well-being or wellbeing):ti,ab,kw

16. MeSH descriptor: [Activities of Daily Living] this term only

17. MeSH descriptor: [Quality of Life] this term only

18. #15 or #16 or #17

19. #5 and #10 and #14 and #18
