# Supplementary material for: Re-thinking reablement strategies for older adults in residential aged care: a scoping review
Source: BMC Geriatr. 2021 Nov 30;21:667. doi: 10.1186/s12877-021-02627-7 (PMC8638477; doi:10.1186/s12877-021-02627-7)
Supplement: Supplementary file 2 — Additional file 2. Summary of included studies. [file 12877_2021_2627_MOESM2_ESM.docx]

**ADDITIONAL FILE 2: SUMMARY OF INCLUDED STUDIES**

| Study | **Participants** | **Intervention / Comparator(s)** | **Outcome measure(s)** | **Main findings** |
| --- | --- | --- | --- | --- |
| (18) | n=29 (IG n=15; CG n=14)  Analysed total n=29    Institutionalised older adults residing in nursing homes  Mean age IG 84.0 (3.0); CG 86.0 (7.5) years.  % female IG 80.0%; CG 85.7% | IG: Whole Body Vibration intervention (3 x week for 8 weeks). Training sessions were supervised by a physiotherapist and one researcher.  CG: Usual nursing home care. | **Physical function**: TUG, 30-s Chair sit to stand (CSTS), postural stability: Kistler force platform, Barthel Index. **QOL**: EuroQoL-5D (mobility, personal care, usual activities, pain / discomfort, and anxiety / depression) | **Physical function.** 8-week follow-up, TUG test (p = 0.002, ES 0.575), 30-s CSTS number of times (p = 0.006, ES 0.515), Barthel Index (p = 0.006, ES -0.515) and Barthel indep (p = 0.004, ES 0.531) improved in the IG compared to the CG. **QOL.** 8-week follow-up, EQ-5Dmobility (p <0.001, ES 0.732) and EQ-5Dutility (p <0.001, ES -0.689) improved in the IG compared to the control.  **Adherence** (IG): 73% (11/15) of all participants completed at least 80% of the sessions. **Adverse events:** None of the participants in the IG reported any adverse health effects during the treatment. |
| (21) | n=112 (IG n=57; CG n=55)  Analysed total n=112  Residents of long-term nursing homes.  Mean age total 84.9 (70-102); IG 85.1 (7.6); CG 84.7 (6.1) years.  % female total 70.5%; IG 73.7%; CG 67.3% | IG: Progressive multicomponent exercise intervention at moderate intensity (2 x week for 24 weeks). Training included individualised strength and balance exercises (60mins). Walking recommendations were provided. All sessions provided by a professional instructor with a degree in physical activity and sport sciences.  CG: Routine low-intensity activities | **Physical function:** SPPB (baseline and 6 months), Barthel Index (baseline and 12 months) | **Physical function**. Lower prevalence of frailty in IG compared to CG (according to SPPB) (67.4% vs 93.0%; p <.05). Significant group-time interaction in favour of IG for reduced frailty (SPPB) (p <.001, η2 = 0.393 large; 95% CI = 0.233-0.518). No significant group-time interaction in the Barthel Index was observed. Significant decline in the CG on the Barthel Index after 12 months (p < .05), whereas the score was maintained in the IG.  **Adherence**: Attendance rates for the exercise sessions were 90.8%, and compliance for the walking recommendations was 79.0%. **Adverse events**: No adverse effects of exercise occurred. |
| (19) | n=112 (IG n=57; CG n=55). Analysed total n=88 (IG n=43; CG n=45)  Residents of long-term nursing homes.  Mean age total 84.9 (70-102); IG 85.1 (7.6); CG 84.7 (6.1) years.  % female total 70.5%; IG 73.7%; CG 67.3% | IG: Progressive multicomponent exercise intervention at moderate intensity (2 x week for 24 weeks). Training included individualised strength and balance exercises (60mins). Walking recommendations were provided. All sessions provided by a professional instructor with a degree in physical activity and sport sciences.  CG: Routine low-intensity activities. | **QOL**: Quality of Life in Alzheimer's Disease rating scale (baseline and 6 months). **Mental health**: Goldberg Anxiety and Depression Scale (baseline and 6 months) | **QOL**: No significant group-by-time interaction for QOL-AD. **Mental health**: No significant group-by-time interactions were found for anxiety or depression.  **Adherence**: Attendance rates for the exercise sessions were 90.8%, and compliance for the walking recommendations was 79.0%. **Adverse events**: no adverse effects of exercise occurred. |
| (20) | n=112 (IG n=57; CG n=55). Analysed total n=92 (IG n=45; CG n=47)  Residents of long- term nursing homes.  Mean age total 84.9 (70-102); IG 85.1 (7.6); CG 84.7 (6.1) years.  % female total 70.5%; IG 73.7%; CG 67.3% | IG: Progressive multicomponent exercise intervention at moderate intensity (2 x week for 24 weeks). Training included individualised strength and balance exercises (60mins). Walking recommendations were provided. All sessions provided by a professional instructor with a degree in physical activity and sport sciences.  CG: Routine low-intensity activities. | **Physical function**: SPPB, 30-s chair stand test, 8-ft TUG, 6MWT, fast 4-m walking speed, Berg balance scale, habitual PA (accelerometer-derived) (baseline and 3 months) | **Physical function**: significant group-time interaction in favour of IG in SPPB score (p < 0.001, η2 = 0.245 medium), 30-s chair stand (p <.001, η2 = 0.131 medium), 8ft-TUGT (p = 0.025, η2 = 0.055 small), fast 4m walking speed p = 026, η2 = 0.055 small), and Berg balance scale (p <.001, η2 = 0.130 small). No significant group-time interactions for 6mWT or habitual physical activity. ** results are also reported separating into groups with low and high physical function at baseline** The significant results for the entire sample above are also significant for the low physical function at baseline sample. The high function group only demonstrated significant differences in SPPB and Berg balance score.  **Adherence**: Attendance rates for the exercise sessions were 90.6%, and compliance for the walking recommendations was 92.0%. **Adverse events**: no adverse effects of exercise occurred. |
| (22) | n=62 (IG n=31; CG n=31).  Analysed total n=55 (IG n=30; CG n=25).  Nursing home residents.  Mean age total 83.2 (7.9); IG 82.2 (9.0); CG 84.2 (6.8) years.  % female: total 76.0%; IG 64.5%; CG 87.1%. | IG: Whole Body Vibration (3 x week for 12 weeks) with exercises. Physiotherapist and researchers supervised the training.  CG: the control group was requested neither to change their lifestyle during the study nor to get involved in any new type of physical activity. | **Physical function**: TUG 3m, walking speed over 20m, Tinetti test (baseline and 3 months). | **Physical function**: no significant difference between groups for walking speed, TUG or Tinetti test (balance and gait).  **Adherence**: 91.9% of the exercise sessions were performed. **Adverse events**: participants in the IG (n=2) experienced hip pains which seemed to be related to the training |
| (23) | n=89 (IG n=22; NMES n=22; NMES+ n=22; control n=23).  Analysed total n=89  Older adults in a nursing home.  Mean age: IG 85.5 (4.7); NMES 82.9 (4.3); NMES+ 83.6 (3.6); CG 83.6 (5.6) years.  % female: IG 68.1%; NMES 63.6%; NMES+ 63.6%; CG 65.2%. | IG: Volitional contraction (**VC**) (3 x week for 16 weeks). Knee extension exercises in both legs with weights 3 x 15 reps, rest periods, 40% 1RM. Each session lasted 30-35 mins. Supervision of two physiotherapists.  CG: Neuromuscular electrical stimulation **(NMES);** Neuromuscular electrical stimulation superimposed onto voluntary contractions **(NMES+);** no intervention (**control**). | **Physical function**: TUG 3m, 6-min walk test, Barthel Index, Berg balance scale (baseline and 4 months). | **Physical function**: significant group-time interaction for TUG (p = .022, ηp2 = .155), and Barthel Index (p = .05, ηp2 = .116). NMES+ showed significant improvement for TUG, no change in VC or NMES, and significant decrease in the control. All intervention groups (VC, NMES, NMES+) significantly improved on Barthel Index, but not the control. No significant group-time interaction for Berg balance scale or 6-min walk test.  **Adherence**: 78% of participants in the intervention groups attended to complete the 48 planned sessions during the 4-months intervention period. **Adverse events**: no significant adverse events were reported by any of the participants. |
| (24) | n =24 (IG n=14; CG n=10). Analysed total n=17 (IG n=13; CG n=4)  Nursing home residents.  Mean age: total 83.7 (6.4); IG 83.6 (7.3); CG 83.8 (5.7) years.  % female: total 87.5%; IG 85.7%; CG 90.0%. | IG: Multicomponent exercise intervention (2 x week for 16 weeks). Included dual task, dynamic balance, endurance and strength exercises. Each session was 45-60min and administered by certified exercise therapist  CG: Waitlist control. | **Physical function**: SPPB, gait performance (step length, width and speed on a 7.92m track), Barthel Index (baseline and 16 weeks). **QOL**: Health Survey (SF-12), Satisfaction with Life Scale (SWS) (baseline and 16 weeks). | **Physical function**: no significant difference between groups for SPPB, gait performance or Barthel Index. **QOL**: No significant difference between groups for SF-12 or SWS.  **Adherence:** The mean participating rate was 12.5±1.9 participants per session. **Ability to perform the intervention** a total of 45 min training duration was feasible for this target group. **Acceptability** Most participants agreed that the program was an overall positive experience. Nursing personnel rated the acceptability of the intervention as high. **Adverse events** There were no adverse events related to the intervention reported. Some muscles soreness reported. |
| (25) | n=123 (n=41 each group).  Analysed total n=109 (combined group n=37; aerobic group n=36; social group n=36)  Institutionalized patients with dementia in a psychogeriatric nursing home.  Mean age: combined group 85.7 (5.1); aerobic group 85.4 (5.4); social group 85.4 (5.0) years.  % female: combined group 78.4%; aerobic group 77.8%; social group 69.4%. | IG: **Combined group** (4 x week for 9 weeks). Alternated strength and walking sessions.  CG**: Aerobic group:** 4 moderate to high-intensity walking sessions per week. **Social group**: 4 one-on-one social visits per week. | **Physical function**: 6-minute walk test, 30-s sit-to-stand test, TUG, Balance; Frailty and Injuries Cooperative Studies of Intervention Techniques-Subtest 4, the figure of eight test, Groningen Meander Walking Test (baseline, 9 and 18 weeks). | **Physical function**: post intervention: combined group improved compared with the social group on 6-min walking test (p = 0.004), 30-s sit-to-stand (p<0.001) and balance (p = 0.002). Combined group scored higher than aerobic group on 6-min walking test (p = 0.004) and 30-s sit-to-stand (p=0.001). After 18 weeks no significant differences between groups remained.  **Adherence**: attendance for the combined group, aerobic group and social group were 89.2%, 89.1%, 93.2%, respectively. |
| (26) | n=62 (n=31 each group).  Analysed total n=43 (IG n=26; CG n=17).  Healthy nursing home residents.  Mean age: total 83.2 (7.9); IG 82.2 (9.0); CG 84.2 (6.8) years.  % female: total 76%; IG 64.5%; CG 87.1%. | IG: Whole Body Vibration (3 x week for 24 weeks) with exercises. All training sessions were supervised by a physiotherapist or a researcher.  CG: no intervention, requested not to change lifestyle or engage in any new type of physical activity. | **Physical function**: TUG 3m; 20m gait test (Locometrix), Tinetti test (baseline, 6 and 12 months). | **Physical function**: at 6 months no significant difference between groups on TUG, Tinnetti test or gait parameters except for step length (decreased in both groups at 12 months but more in the control group (p<0.01)).  **Adherence**: 23 participants completed the intervention and attended 90.9% of the exercise sessions. |
| (27) | n=32 (n=16 each group).  Analysed total n=24 (IG n=11; CG n=13).  Frail nonagenarians residing in a nursing home.  Mean age: total 91.9 (4.1); IG 93.4 (3.2); CG 90.1 (1.1) years.  % female: total 70%. | IG: Multicomponent exercise program (2 x week for 12 weeks). Resistance training combined with balance and gait retraining exercises that progressed in difficulty (40 minutes). All training sessions were supervised by one experienced physical trainer.  CG: Performed mobility exercises (small active and passive movements) 30 min per day, at least 4 days per week. | **Physical function**: TUG 3m, 5-m habitual gait, rise from a chair test, FICSIT-4 tests of static balance, Barthel Index (baseline and 12 weeks) | **Physical function**: significant group-time interaction for 5-m habitual gait velocity (p<0.05), TUG (p<0.01), rise from a chair (p<0.01), and balance (p<0.05). Significant decrease in the 5m habitual gait velocity in CG (p<0.05), no change in IG. IG spent significantly less time on the TUG (p<0.05), whereas a trend toward significantly higher time was observed in CG. IG increased performance in the rise from a chair test (p <0.01), no change observed in CG. Balance improved in IG (although not significantly p>0.05) compared to CG. After training, IG showed significantly lower deterioration in the BI (p<0.001) compared with the CG.  **Adherence**: Adherence of more than 90% was observed in all subjects. |
| (28) | n=78 (IG: n=38; COM+J n=40)  Analysed total n= 66 (COM n=30; COM+J n=36).    Older people living in long term care facilities.  Mean age: COM 81.5 (6.3); COM+J 79.4 (5.4) years.  % female: COM 66.6%; COM+J 55.5%. | IG: Combined exercise program (**COM)** (3 x week for 6 weeks). Exercise program (45minute sessions) included stretching, strength and aerobic exercises Performed under the supervision of a sport teacher and a physiotherapist.  CG: COM + jumping (**COM+J**). Same combined exercised program with additional 10minutes of jumping (self-paced). | **Physical function**: Berg balance test (BBS) (baseline and 6 weeks). **QOL**: Short Form 36 (SF36) (baseline and 6 weeks). **Mental health**: Geriatric Depression Scale (GDS) (baseline and 6 weeks). | **Physical function**: at 6 weeks: improvements in Berg balance score were statistically better (p = 0.02) in the COM+J group in comparison to the COM group. **QOL**: at 6 weeks: no significant difference between groups on SF-36. **Mental health**: at 6 weeks: no significant difference between groups on GDS.  **Adverse events**: None of the subjects were injured. |
| (30) | n=26 (13 each group).  Analysed total n=24 (IG n=11; CG n=13).  Nonagenarian women in a geriatric residential care home.  Mean age: total 90.6 (4.4); IG 90.1 (4.0); CG 91.0 (4.8) years.  % female: total 100%. | IG: Muscle strength training with TheraBands (2 x week for 12 weeks).  CG: Maintain daily routines. | **Physical function:** Barthel Index, 5x sit-to-stand test (FTSTS), TUG (baseline and 12 weeks) | **Physical function**: significant between group difference on the BI (p<0.05) and TUG (total time, speed, m/s) (p<0.05). IG maintained scores on BI and TUG, and CG showed a significant decrease (p<0.05). Significant between group difference on the FTSTS (p<0.05); IG (p<0.005) significantly increased performance on FTSTS, and CG (p<0.05) significantly decreased performance. |
| (29) | n=189 (IG n=73; CG n=116).  Analysed total n=189.  Elderly home care residents with dementia.  Mean age: IG 80.6 (8.3); CG 82.9 (7.4) years.  % female: IG 81.0%; CG 43.8%. | IG: Aerobic physical activity program (7 x week for 60 weeks). Cycled daily alone, or in pairs on a recumbent bike 15mins. Physiotherapists supervised the program.  CG: Usual activities | **Physical function**: TUG, Katz Index of Independence in Activities of Daily Living (baseline and post-intervention). **Mental health:** Cornell Scale for Depression in Dementia (baseline and post-intervention). | **Physical function**: significant between group differences pre-post intervention for TUG test (p = 0.03) and Katz Index (p = 0.03); significant improvements in IG when compared to the CG. **Mental health**: No significant difference between groups for depression.  **Adherence**: individuals in the IG exercised for a mean of 108.45 ± 7.99 min per week and the mean attendance rate was approximately 88%. **Adverse events**: no adverse events attributable to the exercise program. |
| (38) | n=81 (n=27 each group)  Analysed total n=37 (PMTG n=11; RMTG n=9; Control n=17).  Older adults with sarcopenia residing in nursing homes.  Mean age: PMTG 82.6 (9.1); RMTG 87.1 (3.8); Control 81.2 (5.4) years.  % female: PMTG 82%; RMTG 56%; Control 71%. | IG: Peripheral muscle training group (**PMTG**) (3 x week for 12 weeks). Supervised by physiotherapists and completed in small groups (8-10 participants).  CG: Respiratory muscle training group **(RMTG).** Supervised by physiotherapists and completed in small groups (8-10 participants). **Control.** Maintain usual care and daily life activities. | **Physical function**: gait speed 14m (baseline and 12 weeks). | **Physical function**: gait speed remained unchanged from pre-to post intervention in the three groups.  **Adverse events**: Main complaints reported during training were keeping lips constrained in the mouthpiece of the Inspiratory Muscle training device, nasal discomfort from nose clip placement (RMTG), and mild muscular discomfort and generalized fatigue (PMTG). |
| (71) | n=60 (IG n=30; CG n=30).  Analysed total n=60  Elderly long-term care facility residents in wheelchairs.  Mean age total 81.2 (65-95); IG 80.7 (9.6); CG 81.7 (6.3) years.  % female total 63.3%; IG 63.3%; CG 63.3%. | IG: Seated Tai Chi exercise conducted by a qualified Tai Chi instructor (3 x week for 26 weeks).  CG: Usual standard care. | **QOL**: WHOQOL-BREF instrument (Chinese version) (baseline, mid-point at 13 weeks and post-intervention at 26 weeks). **Mental health**: Geriatric Depression Scale-Short Form (Chinese version) (GDS-SF) (baseline, mid-point at 13 weeks and post-intervention at 26 weeks). | **QOL**: At 13 weeks no significant between group difference in overall QOL. At 26 weeks significant between group difference for overall QOL (p= 0.03); in comparison with those in CG, those in IG registered higher scores. No significant difference between groups in the change in mean scores from baseline to week 13 and week 26 for overall QOL. **Mental health**: At 13 weeks, no significant difference between groups for the GDS-SF. At 26 weeks, significant difference between groups for the GDS-SF (p<.005); participants in the IG had lower depression symptom scores than those in the CG. No significant difference between groups for change in mean scores from baseline to 13 weeks for GDS-SF. A significant between group difference for change in mean scores from baseline to week 26 was found for GDS-SF scores (p<0.00); those in IG were 5.2 times more likely to show a reduction in GDS-SF scores than those in CG.  **Adherence**: attendance rate of 85.2% (IG) and 93.2% (CG). In the IG 76% of participants attended more than 80% out of the total Tai Chi sessions. |
| (31) | n=276 (IG n=139; CG n=137) Analysed total n=222 (IG n=104; CG n=118).  Residents of residential aged care facilities.  Mean age: IG 83.9 (6.5); CG 85.3 (5.1) years.  % female: IG 87.5%; CG 86.4%. | IG: Low intensity exercise program (1 x week for 20 weeks) targeting coordination, balance, strength, endurance, sensorimotor perception, breathing, abilities and skills for managing ADL, and interpersonal skills. Session were 60mins performed in groups. Trainers were staff members of facility and supervisors were physical or occupational therapists. A 'home exercise program' comprising 10 exercises was added.  CG: 3 social animation events with no specific focus on physical activity or associated skills. | **Physical function**: TUG, Chair sit and reach test, The Canadian Occupational Performance Measure (baseline and post-intervention). **QOL**: Euro Quality of Life-5 dimensions (EQ-5D) (baseline and post-intervention). | **Physical function**: No significant group-time interaction for TUG, Chair sit and reach test or COPM-performance (per-protocol analyses). **QOL**: significant group-time interaction was found for EQ-5D visual rating scale (p=0.003, ηp2 =0.033); increased in the IG (p=0.001) and showed a negative non-significant change in CG (intention to treat analyses).  **Adherence:** Average participation rate of 58.5% in the IG. **Adverse events**: no unexpected adverse events or harm. |
| (32) | n=191 (IG n=91; CG n=100).  Analysed total n=191.    Older adults dependent in ADLs and living in residential aged care.  Mean age: total 84.7 (6.5, 65-100); IG 85.3 (6.1); CG 84.2 (6.8) years.  % female: total 73%; IG 74%; CG 72%. | IG: High intensity functional weight-bearing exercise (5 x fortnight for 13 weeks). Each session was 45mins and was supervised by two physiotherapists and one occupational therapist.  CG: activities performed while sitting (watching films, singing, reading and conversation). Performed in small groups, 45mins, 5 sessions every two-week period over 13 weeks. | **Mental health**: The Geriatric Depression Scale 15-item version (GDS-15); Philadelphia Geriatric Center Morale Scale (PGCMS) (baseline, 3 and 6 months). | **Mental health**: no between group differences in GDS or PGCMS scores at 3 or 6 months. Among participants with dementia there was a significant between group difference in PGCMS at 3months in favour of the IG (p = 0.03).  **Adherence**: attendance 72% in IG and 70% in CG. **Adverse events**: no adverse event led to any injury or disease. No significant differences were observed in applicability (attendance, achieved intensity, adverse events) when comparing participants with and without dementia. |
| (33) | n=160 (IG: n=51; CA n=49; Control n=60).  Analysed total n=160.  Highly deconditioned, elderly nursing home and long-term care home residents.  Mean age: total 82.3 (9.1, 65-102); IG 83 (8.6); CA 83.2 (8.3); Control 80.9 (10.1) years.  % female: total 71.7%; IG 76.5; CA 63.3%; Control 75.0%. | IG: Adapted tai chi program (**AT**) (4 x week for 24 weeks). Classes taught by experienced instructor. 30min sessions in small groups (8 participants).  CG: **Cognition-action (CA)** program. Warm-up, lower limb movements alternated with upper body exercises followed by stretching and resistance exercises against the hand of instructor. 2x 30-40 min sessions per week for 6 months monitored by trained physical activity teacher. **Control**: Usual care. | **Physical function**: Katz and colleagues index of ADL, TUGT 3m, Chair rise test, 10m walking speed (baseline, 6 and 12 months). **Mental health**: Geriatric Depression Scale (baseline, 6 and 12 months). | **Physical function**: at 6 (p<.001) and 12 months (p=.007) significant difference in ADLs between groups. At 6 months control experienced a significant decline in total ADL score (p<.001), no significances change in AT or CA groups. At 12 months the control had a larger decline in total ADL score compared with AT and CA groups. The change in total ADL score over 12 months was not significantly difference between CA group and control or between AT group and control. At 6 (p<.001) and 12 months (p=.001) significant between group difference on chair rise test. At 6 months control significantly worsened on chair rise test (p<.001), no change in AT or CA. At 12 months CA and control improved on chair rise test and AT declined (changes not significant). At 6 (p=.04) and 12 months (p=.04) significant between group difference for 10m walking speed. At 6 months control performance significantly worsened (p<.001), no change in IG. At 12 months, walking speed significantly declined in all groups (AT group, p=.03; CA group, p=.02; control p<.001). No significant between group differences on TUG at 6 or 12 months. **Mental health**: at 6 months no between group difference on GDS. At 12 months significant between group difference on GDS (p=.008), CA significantly reduced depressive symptoms (p<.001), no change in AT or control.  **Adherence**: attendance rates for the 6-month period were similar in both exercise groups (48.9% in the CA group and 38.8% in the AT group). **Adverse events**: none of the observed deaths (n=18) was directly or indirectly attributable to an adverse effect due to the intervention |
| (64) | n= 30 (IG n=18; CG n=12). Analysed total n=25 (IG n=14; CG n=11).    Older adults with mild to moderate cognitive impairment residing in an assisted living facility.  Mean age: total 86.5 (5.3); IG 87.6 (5.5); 85.4 (5.0) years.  % female: total 53%; IG 50%; CG 55%. | IG: Interrupting sedentary behaviour and substituting it with a 10-min bout of light physical activity (walking) (3 x day for 10 weeks). Participants provided with a digital sports watch that vibrated (15-s vibration) at three different times throughout the day (30min after each meal), prompting participants to engage in a 10-min bout of physical activity.  CG: Regular daily activities | **Physical function**: TUG, Actical accelerometer (PA) (baseline and post-intervention). **QOL**: 36-Item Short-Form Health Survey (SF-36) (baseline and post-intervention). | **Physical function**: significant time-group interaction for mins spent in light PA (p<.001, ηp2 =.53) and moderate PA (p=.006, ηp2 =.3). IG increased their light and moderate PA more than the CG. Significant time-group interaction was found for the TUG (p <.001, ηp2 =.5); IG improved TUG times compared with the CG. **QOL**: Significant time-group interactions on components of the SF-36 favouring the IG; physical functioning (p=.005, ηp2 =.30), role limitations due to physical health (p=.003, ηp2 =.3), general health (p=.001, ηp2 =.4), social functioning (p=.029, ηp2=.19), energy (p<.001, ηp2=.6), mental health (p<.001, ηp2=.4), role limitations due to emotional health (p<.001, ηp2 =.5). No significant time-group interaction on pain component.  **Adherence**: IG had 100% retention throughout the 10 weeks. 10/14 participants in IG documented completing over 80% (17/21) of the PA bouts and 13/14 documented completing at least 70% (15/21) of the bouts. **Adverse events**: none. |
| (34) | n=42 (IG n=21; CG n=21).  Analysed total n=42.  Institutionalized older adults residing in a nursing home.  Mean age: IG 83.2 (6.5); CG 82.7, (6.4) years.  % female: IG 66.7%: CG 71.4%. | IG: Multicomponent intervention (5 x week for 12 weeks). Received both physical therapy program (3 x week 45 minutes) and proprioceptive exercise program (2 sessions per week 55 minutes).  CG: Received physical therapy program. 3 sessions per week, for 12 weeks. | **Physical function**: TUG, Tinnetti test, Cooper test (baseline and post-intervention). | **Physical function**: significant time-group interaction for TUG (p=.002), Tinnetti test (p=.015) and Cooper test (p=.019). IG demonstrated significant improvements on the TUG (p<0.001) and Tinetti test (p=.041), no change in CG. IG demonstrated significant improvement on Cooper test (p=.024), CG significant declined (p=.009). |
| (65) | n=45 (IG n=15; CG n=30).  Analysed total n=37 (IG n=13; CG n=24).  Institutionalized frail elderly in a long term care facility.  Mean age: 76.1 (7.7); IG 73.3 (6.4); CG 77.8 (8.0) years.  % female: not reported. | IG: Multicomponent exercise program (3 x week for 12 weeks). Exercises were focused on improving mobility, flexibility, strength and aerobic resistance. Performed in small groups, followed by 2 physical educators and 3 monitors. Each session was 40mins.  CG: Usual activities. | **Physical function**: TUG 3m; Sitting and Lift Test, Katz scale, Fried et al criteria (frailty) (baseline and post-intervention). **Mental health**: Yesavage scale (depression) (baseline and post-intervention). | **Physical function**: At 12 weeks significant between group difference on the TUG (p<0.01); IG performed significantly better than CG. At 12 weeks significant between group difference on Katz scale (p<0.05); IG significantly lower dependence in ADLs than CG. No significant between group different on the Sitting and Lift test at 12 weeks. The IG showed a significant (p<0.05) ~34% reduction in prevalence of criteria for frailty, while CG showed a reduction of ~6%. A significant (p<0.05) decrease of more than ~73% in the number of elderly classified as frail in IG (most moving to prefrail status), CG remained similar. **Mental health**: At 12 weeks no significant between group difference in depression.  **Adherence**: 61.5% attendance at exercise sessions. |
| (35) | n=322 (IG n=170; CG n=152).  Analysed total n= 241 (IG n=129; CG n=112).    Nursing home residents.  Mean age: IG 85.0 (7.9); 84.5 (7.3) years.  % female: IG 70.5%; CG 79.0%. | IG: Individually tailored program (daily for 12 weeks) consisting of physical and daily activities in different combinations, depending on goals and physical and cognitive functions of each participant. Physiotherapist and occupational therapist were responsible for the intervention.  CG: Ordinary care. | **Physical function**: Functional Independence Measure (FIM); Berg Balance Scale (BBS); Timed Chair Stand Test; Nursing Home Life Space Diameter (NHLSD; physical activity), walking/wheelchair propulsion 10m (self-selected speed and maximum speed), Clinical Outcome Variable Scale (COVS) (transfers) (baseline and 6 months). **Mental health**: Philadelphia Geriatric Centre Morale Scale (PGCMS) (baseline and 6 months). | **Physical function**: significant between group difference in mean change from baseline to 6 months in transfers (COVS, p=0.04) in favour of the IG; CG deteriorated significantly (p=0.006), IG remained stable. Significant between group difference in mean change from baseline to 6 months in FIM social and cognitive function subscale (p=0.03); IG deteriorated in social and cognitive function (p=0.004), CG remained stable. No significant difference between the IG and CG in mean change from baseline to 6 months on FIM (physical function subscale), BBS, Timed Chair Stand Test, NHLSD, or Walking/ wheelchair propulsion. **Mental healt**h: no significant difference between the IG and CG in mean change from baseline to 6 months on the PGCMS.  **Adherence**: 74 participants took part in 10-13 weeks of the intervention, mean 93min per week. The most common intervention was focused on decreasing activity limitation (e.g. transfers, walking and personal care). **Adverse events**: no major adverse events. |
| (36) | n=322 (IG n=170; CG n=152)  Analysed total n= 322    Nursing home residents.  Mean age: IG 85.0 (7.7); CG 84.9 (7.6) years.  % female: IG 71.0%; CG 76.0%. | IG: Individually tailored program (daily for 12 weeks) consisting of physical and daily activities in different combinations. Physiotherapist and occupational therapist were responsible for the intervention.  CG: Ordinary care. | **Physical function**: Functional Independence Measure (FIM); Berg Balance Scale (BBS); Timed chair stand test; Nursing Home Life Space Diameter (NHLSD; 'semi total' extension of PA; 'total' subscale extension and dependency in PA), walking/wheelchair propulsion 10m (self-selected speed and maximum speed), COVS (transfers) (baseline and 3 months). | **Physical function**: at 3 months significant difference between groups in balance (p=0.001), physical activity (NHLSD semi total subscale) (p=0.029) and transfers (p =0.012); IG had improved while the CG had deteriorated. No significant between group difference for ADL, physical activity (NHLSD total subscale), Timed chair stand test, 10m indoors walking or wheelchair propulsion.  At 3 months those who had taken part in 150min or more of intervention per week had significantly better balance (p=0.003), higher transfer scores (p=0.046) and were more physically active (NHLSD total and semi total) (p=0.005, p=0.001) than the others.  **Adherence**: 68% completed 10-13 weeks of intervention with a mean dosage of intervention of 117min per week. Decreasing activity limitations (53.0%) and functional impairments (32.2%) was the focus of majority of interventions. **Adverse events**: no major adverse events. |
| (66) | n=16 (IG n= 8; CG n=8).  Analysed total n=16.    Older adults with dementia residing in long term care facilities.  Mean age: IG 83.2 (4.3); CG 82.8, (7.8) years.  % female: total 63%. | IG: Walking program (3 x week for 12 weeks). Walking at self-selected pace in small groups for 15mins or until they reached moderate breathlessness. Walking time was increased by 5mins bimonthly or as tolerated by each group. Walking program led by volunteers (from university community).  CG: Social activity, received weekly visits (30-45mins) from a volunteer. Visits based around sedentary activity that participant enjoyed. | **Physical function**: TUG, six-minute walk test (6MWT), functional reach test (FR; balance) (baseline, 6 and 12 weeks). | **Physical function**: no significant group-time interactions on TUG, 6MWT, or FR.  **Adherence**: compliance over the 12 weeks of the intervention was high (IG 74%; CG 80%, no sig diff). |
| (37) | n=52 (IG n=27; CG n=25).  Analysed total n=52    Low-functioning older individuals living in residential care facilities.  Mean age: total 81.9 (8.6); IG 81.0 (9.6); CG 82.8 (7.5) years.  % female: total 88%; IG 92%; CG 84%. | IG: Dance-based exercise program (EXDASE) (1 x week for 12 weeks). Program consisted of 75min group sessions; single instructor administered all sessions.  CG: Regular facility activities. | **Physical function**: Senior fitness test; 30-s chair stand test, 2-minute step test, chair sit-and-reach test, TUG (baseline and post-intervention). | **Physical function:** significant group-time interactions in favour of the IG for 30-s chair stand test (p<.001), 2-min step test (p=.009), the chair sit-and-reach test (p =.026) and the TUGT (p=.014); IG outperformed CG with respect to pretest-to-post-test changes in performance.  **Adherence**: most participants attended more than 75% of the sessions, and no one completed fewer than 50% of sessions. **Adverse events**: no injuries were reported due to the intervention. |
| (77) | n= 60 (n=30 each group)  Analysed total n=60    Older people living in a long-term care facility and using wheelchairs for mobilisation.  Mean age: total 81.2 (7.9; 65-95); IG 80.7 (9.6); CG 81.7 (6.3) years.  % female: total 63.3%; IG 63.3%; CG 63.3%. | IG: Seated Tai Chi exercise program (3 x week for 26 weeks) conducted by a qualified Tai Chi instructor.  CG: Usual activities. | **Mental health**: Profile of Mood States Short Form (POMS-SF) (baseline, 13 weeks and post-intervention). | **Mental health**: no significant between group differences were found in overall mood state (POMS-SF) at 13 and 26 weeks. No significant between group difference in change in mean scores from baseline to week 13 and week 26 for overall mood state. |
| (39) | n=45 (MT n=23; FWT n=22). Analysed total n=29 (MT n=14; FWT n=15).    Institutionalized older adults residing in a nursing home.  Mean age: total 84.1 (8.3); MT 78.9 (9.1); FWT 89.0 (2.8) years.  % female: total 69.0%%; MT 57.1%; FWT 80%. | IG: Machine training group **(MT)** (2 x week for 12 weeks). 45-60 mins sessions performed in small groups. Performed under supervision by 2 exercises specialists.  CG: Free weight training group (**FWT)**. 2 weekly non-consecutive sessions for 45-60mins for 12 weeks. Performed in small groups under supervision of exercise specialist. | **Physical function**: TUG, 30-s Chair Rise Test, 10-m walk test, 11-step stair climbing test (baseline and post-intervention). | **Physical function**: no significant group-time interactions for TUG, 30-s Chair Rise Test, 10-m walk test or 11-step stair climbing test. Performance on 30-s chair rise test improved significantly in both MT and FWT groups (p=0.033); FWT revealed higher values than MT.  **Adherence**: Participants attended 91.1 and 83.9% in the FWT and MT groups, respectively. **Adverse events**: no adverse effects. |
| (40) | n=77 (IG n=38; CG n =39).  Analysed total n=60 (IG n=27; CG n=33).    Independently walking, cognitively unimpaired nursing home residents.  Mean age: total 78.4 (7.6, 65-90); IG 78.3 (8.1); CG 78.5 (7.2) years.  % female: total 66.2%; IG 73.7%; CG 59.0%. | IG: Otago exercise program (3 x week for 24 weeks). Individually tailored, strength and balance program in groups. Supervised by physiotherapists.  CG: Advised to continue with standard care and activities. | **Physical function**: BBS, TUG 3m, chair rising test, motor Functional Independence measure (mFIM) (four areas of ADLs: self-care activities, sphincter control, transfers and locomotion) (baseline, 3 and 6 months). | **Physical function**: significant group-time interactions for BBS (p<0.001), TUG (p<0.001), chair rising test (p<0.001) and mFIM (p=0.019). In the IG performances significantly improved and in the CG performance was maintained (BBS, TUG, mFIM) or significantly worse (CRT).  **Adherence**: participants in the IG adhered to 92.7% of 78 training sessions through 6 months (mean 72 out of 78 sessions). **Adverse events**: no major adverse events were reported. Fatigue and muscle soreness were reported during first 7 weeks in IG. |
| (41) | n=86 (IG n=43; CG n=43).  Analysed total n=86.  Older persons with cognitive impairment living in a long term care institute.  Mean age: IG 76.3 (9.6); CG 79.2 (12.6) years.  % female: IG 83%; CG 79%. | IG: Multimodal exercise program (2 x week for 48 weeks) delivered by physiotherapists.  CG: Usual care. Participating in activities such as simple board games, viewing pictures of films, listening to music, arts and crafts activities: embroidery, needlework, conversations | **Physical function**: Performance Oriented Mobility Assessment (POMA) scale (static and dynamic balance), TUG-3m, Katz Index (baseline, 6 and 12 months). | **Physical function**: significant change in POMA balance score over time in the IG (p<0.0001) but not in the CG. POMA balance score increased significantly at both 6 (p<0.0001) and 12 months (p=.002) in IG. Significant change in POMA gait score over time in the IG (p<0.0001) but not in the CG. POMA gait score increased significantly at 12 months (p=.001) in IG. Significant change in TUG time in the IG (p<0.0001) but not in the CG. TUG time improved at 12 months (p=0.004) in IG. No significant between group difference in Katz Index. |
| (72) | n=73; WBV + exercise n=25; exercise n=24; control n=24  Analysed total n=73  Institutionalized older adults residing in nursing homes.  Mean age: total 82.3 (7.3); WBV + exercise 84.0 (6.7); exercise 82.4 (7.6); control 80.3 (7.3) years.  % female: total 54.7%; WBV + exercise 52%; exercise 58.3%; control 54.1%. | IG: WBV + exercise group (3 x week for 8 weeks). Strength and balance program combined with WBV. Training sessions were supervised by a physiotherapist and nursing home worker.  CG: **Exercise group**. Identical exercise program without WBV. **Control group**. Engaged in social and recreational activities that only involved upper limbs. | **Physical function**: TUG, BBS, 5 times sit-to-stand test, 6-min walk test (baseline and 8 weeks). | **Physical function**: no significant group-time interaction for TUG, BBS or 6-min walk test. Significant group-time interaction was identified for 5 times sit-to-stand test (p=0.048). The exercise group improved performance on the 5 times sit-to-stand test compared to decline in control group (p=0.030).  **Adherence:** no significant difference among the three groups in attendance rate; WBV + exercise 77.1%, exercise 67.5% and control 74.5%. **Adverse events**: no adverse events were reported. |
| (67) | n=42; IG n=28; IG n=14 Analysed total n=33 (IG n=21; CG n=11).  Older adults residing in assisted living residences.  Mean age: IG 80.1 (7.5); CG 83.2 (6.7) years.  % female: IG 71%; CG 91%. | IG. Exercise program using gerotechnology (2 x week for 12 weeks). Kinesiologist supervised sessions at beginning of the intervention.  Control group. | **Physical function**: Functional Autonomy Measurement System (SMAF), Study Osteoporotic Fractures Index (SOF; frailty), TUG 3m, SPPB, chair test, walking speed, Rapid Assessment of Physical Activity (RAPA) (baseline, 12 and 24 weeks). **QOL**: Short Form Health Survey (SF-36) (baseline, 12 and 24 weeks). | **Physical function**: at 12 weeks, significant between group difference for change in physical activity level (p=0.01) and walking speed (p=.03); IG increased physical activity and walking speed compared to CG.  At 24 weeks, significant between group difference for change in Frailty index (p=.03), walking speed (p=.04), TUG (p=.04) and SPPB score (p=0.3). IG improved walking speed, TUG performance, SPPB score and showed reduced frailty status compared to CG. No significant difference between groups for changes on functional autonomy or chair test between baseline and 12 weeks and baseline and 24 weeks. **QOL**: no significant difference between groups for changes on SF-36 between baseline and 12 weeks and baseline and 24 weeks.  **Adherence:** IG completed an average of 89.2% of the prescribed exercise sessions. **Acceptability:** the exercise program was found to be acceptable by participants in terms of difficulty (92.8% of exercises rated as easy or a little difficult) and enjoyment (93.8% of exercises rated as being enjoyed). **Adverse events:** 2 adverse events (falls) occurred but did not lead to any serious injury. |
| (73) | n=69; strength group n=35; aerobic group n=34  Analysed total n=61 (strength group n=30; aerobic group n=31).  Elderly patients with dementia residing in a nursing home.  Mean age: total 85.7 (6.9); strength group 86.7 (6.9); aerobic group 84.6 (6.7) years.  % female: total 18.0%; strength group 20%; aerobic group 16.1%. | IG: Strength training program (5 x week for 4 weeks). Involved the use of isotonic weight training machines targeting the biceps, triceps, pectoralis, gluteus and quadriceps muscles. Sessions were executed under supervision of a certified fitness trainer.  CG: Aerobic training program. Stationary bike training (30min sessions). 5 days per week under supervision of certified fitness trainer. | **Physical function**: Barthel Index (baseline and 4 weeks). **Mental health**: Geriatric Depression Scale (5-item) (baseline and 4 weeks). | **Physical function**: no significant between group difference in the amount of change in the Barthel Index between the strength and aerobic training groups. **Mental health**: no significant between group difference in the amount of change in the GDS-5 between the strength and aerobic training groups. |
| (42) | n=80 (40 in each group)  Analysed total n=80  Elderly individuals living in a nursing home with depressive symptoms.  Mean age: not reported but >65 years.  % female: IG 42.5%; CG 47.5%. | IG: Physical Activity Program (4 x week for 10 weeks) included rhythmic exercises and walking. Participants engaged in the program with the researchers.  CG: No planned implementation of control group. | **QOL**: SF 36 Quality of Life Scale (subdimensions; physical health, physical role, pain, general health perceptions, vitality, social function, emotional role, mental health) (baseline and 10 weeks). **Mental health:** Beck Depression Inventory (BDI) (baseline and 10 weeks). | **QOL**: at 10 weeks, significant between group differences in all subdimensions of the Quality of Life Scale; physical health (p =0.011), physical role (p=0.016), pain (p=0.022), general health perception (p=0.027), vitality (p=0.033), social function (p=0.038), emotional role (p=0.042) and mental health (p=0.044). Mean scores in all subdimensions significantly improved in the IG, no change in the CG. **Mental health**: at 10 weeks significant between group difference in scores on BDI (p =0.005). IG showed significant decrease in the BDI, no change in CG. |
| (80) | Analysed total n=119 (E n=37; SA n=32; ESA n=22; UC n=28).  Residents of nursing homes and assisted living centres.  Mean age: E 81.7 (7.9); SA 80.9 (9.4); ESA 81.0 (9.9); UC 81.9 (6.7) years.  % female: total 64.7%; E 70%; SA 77%; ESA 45%; UC 64%. | IG: High-intensity resistance strength training and walking **(E)** (5 x week for 7 weeks). High-intensity resistance strength training (40mins; 3 x week) and walking (2 x week). Nurse-supervised sessions.  CG: Individualised social activity **(SA).** 5 x week for 7 weeks. Petting a stuffed cat, tossing a ball, or playing checkers. **ESA**. Resistance strength training and walking combined with individualized social activities (7 weeks). **Usual care (UC).** Usual social and physical activities provided in the facility. | **Physical function**: Nursing Home Physical Performance Test (NHPPT) (everyday function) (baseline and post-intervention). | **Physical function**: significant intervention effects on the NHPPT scores among the group (p=0.01), the centred baseline NHPPT covariate (p=0.01), and the group by centred pre-NHPPT interactions (p=0.01). ESA resulted in significantly greater improvements in NHPPT than SA and UC.  **Adherence:** Attendance rates for the E, SA, and ESA groups were 81%, 94% and 80%, respectively. **Adverse events:** Five adverse events were reported (E=4, ESA=1) and included chest-pain (n=2), electrocardiographic changes (n=2) and pain (back, hip, leg, n=1). |
| (43) | n=211; IG n=105; CG n=106.  Analysed total n=189 (IG n=92; CG n=97).  Nursing home residents.  Mean age: total 82.9 (8.5); IG 83.0 (9.1); CG 82.8 (8.1) years.  % female: total 91%; IG 93%; CG 89%. | IG: Dance-based exercise program (EXDASE) (1 x week for 12 weeks).  CG: Wait list control. | **Physical function**: Get-up-and-go test 3m, Barthel Index, Lawton IADL scale, SFT (chair stand test, 2-min step test, chair sit-and-reach test, 8-foot timed test) (baseline and 12 weeks). | **Physical function**: significant group-time interactions for get-up-and-go test (p=0.008) and Lawton IADL (p=0.039); CG declined (p<.05), no change in performance for IG. No significant group-time interaction for Barthel Index. **SFT only assessed in subgroup of participants (n=52; IG n=27; CG n=25); significant group-time interactions for chair stand test (p=0.000), 2-min step test (p=0.009), chair sit-and-reach test (p=0.026), 8-foot timed test (p=0.014). IG improved on chair stand test (p=.006) and CG declined (p=.011). CG declined on 2-min step test (p=.024) and 8-foot timed test (p=.047), no change in performance for IG. IG increased performance on chair sit-and-reach (p=.008), no change in CG. |
| (68) | n=45; IG n=24; CG n=21.  Analysed total n=45.  Institutionalised older adults in long term care facilities.  Mean age: total 84.8 (4.1, 78-89); IG 84.8 (4.1); CG 82.3 (3.2) years.  % female: 100%. | IG: Multisensory exercise program (3 x week for 16 weeks). Program included progressive exercises focused on strength, coordination, balance, multisensory stimulation, and flexibility.  CG: Waitlist control. | **Physical function:** BBS, TUG, Physical Performance Test (baseline and post-intervention). | **Physical function**: significant group-time interaction for BBS (p=.01, ES 0.77), TUG (p =.04, ES 0.51) and Physical Performance Test (p=.01, ES 0.86). The IG significant improved on BBS (p=.02), TUG (p=.01) and Physical Performance Test (p=.04), this was not observed in the CG. |
| (44) | n=20 (n=10 in each group).  Analysed total n=20.  Physically inactive older residents of a nursing home.  Mean age: total 76.7 (8.7, 65-91) years.  % female: total 30%; IG 30%; CG 30%. | IG: Inertial training (2 x week for 6 weeks) using a Cyklotren inertia device. Training conducted by researchers.  CG. Maintained normal daily activity. | **Physical function**: Activities of Daily Living Questionnaire; The Chair Stand Test 30s, 8-Foot Up-and-Go, Tinetti test (gait and balance) (baseline and 6 weeks). | **Physical function**: relative change in performance on chair stand test was significantly different between groups (p<.05); performance significantly increased (40.6%) in IG and remained unchanged in CG. Relative change in performance on 8-F up-and-go test was significantly different between groups (p<.05); time to complete 8-F up-and-go significantly decreased by 12.8% in IG, no significant change in CG. Relative change in Tinetti gait and balance were significantly difference between groups (p<.05); significant improvements in gait and balance in IG (18.6% and 29%,), no significant change in CG. No significant between group difference in relative change in ADLs.  **Adverse events**: no one had an injury or a health problem following training. |
| (45) | n=91; SE n=23; NW n=21; SE+ NW n=23; CG n=24.  Analysed total n=83 (SE n=21; NW n=21; SE+ NW n=21; control n=20).  Osteoporotic and osteopenic females living in residential care facilities.  Mean age: 81 (65-98) years.  % female: 100%. | IG: Modified Sinaki exercises **(SE)** (2 x week for 12-month period). Also received pharmalogical treatment.  CG: Nordic walking **(NW)** (2 x week for 12-month period). Nordic walking sessions were held by a qualified NW instructor. Also received pharmacological treatment. **SE+ NW**. Combination of modified Sinaki exercises and Nordic walking. Also received pharmacological treatment. **Control group**. Pharmacological treatment | **QOL:** QUALEFO-41 (baseline and post intervention) | **QOL**: 2-factor ANOVA with repeated measures identified that both the effects of the particular therapies (p=.00) and the overall effect of the intervention (change pre-post intervention) (p=.00) significantly influenced QOL. Life quality improved in all 3 intervention groups but decreased in control group. Statistically significantly better quality of life in the SE group (p=.01) and SE+ NW group (p<.01). |
| (69) | n=22; IG n=11; CG n=11.  Analysed total n=22.  Subjects with mild Alzheimer’s dementia living in an assisted living facility.  Mean age: IG 79.3 (9.8); CG 81.6 (5.2) years.  % female: IG 72.7%; CG 72.7%. | IG: Wii-Fit intervention (5 x week for 8 weeks). Wii-Fit program included strength training, yoga and balance games. Individual exercise program whereby subjects exercised one on one with help from research personnel.  CG: Walking (5 x week for 8 weeks). Walked at their own pace in small groups at any given time with research personnel. | **Physical function**: Berg Balance Scale (BBS), Tinetti Test (TT), TUG 3m, activities of daily living (ADL), instrumental activities of daily living (IADL) (baseline, 4 and 8 weeks).  **QOL:** Quality of Life-AD (QOL-AD) (baseline, 4 and 8 weeks). | **Physical function:** there were no significant group-by-time interactions on the measures of BBS, TT, TUG 3m, ADLS or IADLs. **QOL**: There was no significant group-by-time interaction on the measure of QOL-AD.  **Adherence:** the total time of participation in the study interventions was not significantly different between the IG and CG. **Acceptability**: the Wii-Fit and walking interventions were well accepted by the participants. Participants reported enjoying the Wii-Fit exercises. **Adverse events**: there were no adverse events. |
| (75) | n=59; IG n=31; CG n=28.  Analysed total n=50 (n=25 in each group).  Ambulation-challenged residents with walking potential in long-term care facilities.  Mean age: IG 75.4 (12.2); CG 78.4 (12.8) years.  % female: IG 36%; CG 44%. | IG: Individualized task-oriented ambulation training program of 30-45 mins per session (3 x week for 4 weeks). Training program was composed mainly of walking-related functional tasks in weight-bearing positions. Physical therapist conducted and tailored exercise tasks for each subject according to the subject’s motor ability.  CG: Usual care. | **Physical function**: Walking speed, TUG 3m, 6-Minute Walk Test (6MWT), Berg Balance Scale (BBS), Barthel Index (BI) (baseline and 4 weeks). | **Physical function**: significant group-time interaction in walking speed (p=0.000), TUG (p=0.005), and BBS (p=0.001). At 4 weeks, walking speed, BBS and TUG significantly improved in the IG as compared with the CG. There were no significant group-time interactions for 6MWT or BI.  **Adherence**: a total of 50 (84.7%) subjects (n=25 in each group) attended every exercise session. |
| (46) | n=85; IG n=43; CG n=42.  Analysed total n=68 (IG n=33; CG n=35).  Older adults living in long term nursing homes.  Mean age: total 84.8; IG 85.3 (7.1); CG 84.9 (6.7) years.  % female: total 68.4%; IG 65.1%; CG 69.1%. | IG: Multicomponent exercise program (2 x week for 12 weeks). The program consisted of individually adapted strength and balance exercises performed at moderate intensity (60mins per session).  CG: Dual-task group. Performed simultaneous cognitive tasks to the same exercises in the multicomponent program. | **Physical function**: Gait speed, Short Physical Performance Battery (SPPB), the Senior Fitness Test (SFT), instrumented Timed Up and Go test (iTUG) (G-WALK triaxial accelerometer and gyroscope), Fried frailty score, SOF index, Tilburg frailty indicator (baseline and post-intervention). **QOL**: Quality of Life Alzheimer’s Disease scale (baseline and post-intervention). **Mental health**: Anxiety and Depression Goldberg Scale (baseline and post-intervention). | **Physical function**: no group-by-time interactions were found for gait speed, SPPB, 6-min walk test, iTUG, Fried Frailty, SOF index or Tilburg frailty indicator. A significant group-by-time interaction was found for the chair stand test (p=0.04); larger improvements for the IG. **QOL**: no group-by-time interactions were found for QOL. **Mental health**: no group-by-time interactions were found for anxiety or depression.  **Adherence**: significant difference in session attendance rates between the IG (91.4%) and CG (84.8%). **Adverse events**: no negative effects were reported. |
| (47) | n=81; IG n=41; CG n=40.  Analysed total n=65 (IG n=32 CG n=33).  Older adults living in long term nursing homes.  Mean age: IG 84.7 (6.5); CG 83.8 (6.2) years.  % female: IG 63.4%; CG 67.5%. | IG: Multicomponent exercise program (2 x week for 12 weeks). The program consisted of individually adapted strength and balance exercises performed at moderate intensity (60mins per session).  CG: Walking program (2 x week for 12 weeks). Research staff walked with participants in small groups. | **Physical function:** SPPB, Senior Fitness Test (SFT; chair stand 30-s, 6MWT), Berg balance scale (BBS), usual gait speed 4m, instrumented Timed Up and Go test (iTUG; BTS Biomedical G-WALK triaxial accelerometer and gyroscope), tri-axial accelerometers (physical activity) (baseline and post-intervention). **QOL**: Quality of Life Alzheimer´s Disease scale (baseline and post-intervention). **Mental health**: Anxiety and Depression Goldberg Scale (baseline and post-intervention). | **Physical function:** there were statistically significant group-by-time interactions in favour of the IG for the SPPB total score (p<.01), sit-to-stand (p<.01), balance (<.05), chair stand 30-s (p<.01), 6MWT (p<.05), the BBS (p < .01). No significant group-by-time interactions for gait speed (SPPB), iTUG, gait speed 4m or physical activity. **QOL**: no significant group-by-time interactions were found for QOL. **Mental health:** no significant group-by-time interactions were found for anxiety of depression.  **Adherence**: significant difference in session attendance rates between the IG (91.9%) and the CG (67.8%). **Adverse events**: no adverse effects were reported. |
| (70) | n=50; by group not reported.  Institutionalised older adults.  Mean age: not reported but > 60 years.  % female: 100%. | IG: Kinect-based exercise (3 x week for 12 weeks). X-Box 360 Console, activities included correspond to balance games, strength tasks, and light aerobic tasks (e.g. walking, dance). Sessions were 60mins.  CG: Played board games and encouraged to continue normal daily activities. | **Physical function:** chair stand test 30s, sit-and-reach test, static balance, 800m walk test, 8-foot up and go (baseline and post-intervention). **QOL:** WHOQOL-BREF (baseline and post-intervention). **Mental health:** Beck Depression Inventory (baseline and post-intervention). | **Physical function**: significant between group difference for time scores on the 8-foot up-and-go test (p<0.001), an improvement of 67% was found in the IG when compared to the CG. Significant between group differences were found for the sit-and-reach test (p<0.001) and balance (p<0.001); increase of 19% on sit-and-reach test and 24% on balance test in IG, no significant change in the CG. Significant between group difference for 800m walk test (p<.001); IG showed a reduction of 55% in time to accomplish task, no difference in CG. Significant between group difference for sit-and-stand test (p<.001); IG experienced an increase in performance on the sit-and-stand test of 21%, no difference in CG. **QOL**: Significant between group difference for QOL, specifically for physical domain (p<0.001), psychological domain (p<0.001), social domain (p<0.001) and environmental domain (p<0.001); significant improvements in IG compared to CG. **Mental health:** significant between group difference in depression (p<0.001); levels of depression were significantly reduced in IG compared with CG. |
| (48) | n=66 (n=22 in each group)  Analysed total n=48 (n=16 in each group)  Institutionalised frail elderly.  Mean age: HI 84.1 (6.8); LI 84.5 (4.8); CG 85.3 (4.7) years.  % female: not reported. | High intensity strength training (3 x week for 8 weeks) **(HI)**. Trained at 70% of one RM; one set of 6–10 repetitions with 6–8s for each repetition. Each training session lasted for 40 min and was supervised by a physiotherapist.  CG: Low intensity strength training **(LI)**. Same training as HI group but trained at 40% of one RM. **Usual care** **(UC)**. | **Physical function:** SPPB, Barthel Index (BI), The Lawton–Brody Instrumental Activities of Daily Living Scale (IADL) (baseline and post-intervention). **QOL**: WHO Quality of Life Instrument-Older Adults Module (WHOQOL-OLD) (baseline and post-intervention). **Mental health:** Geriatric Depression Scale (GDS) (baseline and post-intervention). | **Physical function:** significant between group differences in SPPB (p<0.001), BI (p<0.001) and IADL (p<0.001). At 8 weeks, HI and LI had significantly improved on SPPB (p<0.05)), although SPPB score was significantly better in HI group than LI group (p=0.036), whereas SPPB score had significantly decreased in UC (p<0.05). At 8 weeks, significant improvements in BI and IADl scores in HI and LI groups (p<0.05), however, final scores did not differ between HI and LI groups, no changes in UC. **QOL**: significant between group difference in QOL (p<.001). QOL had significantly improved in HI and LI groups (p<0.05), but no significant difference in WHOQOL-OLD scores between HI and LI groups, significant decline in UC (p<0.05). **Mental health**: significant between group difference in depression (p<0.001). Depression had significantly improved in HI and LI groups (p<0.05), but no significant difference in GDS scores between HI and LI groups, significant decline in UC (p<0.05). |
| (78) | n=33; Tai Chi n=11; Yoga n=11; UC n=11  Analysed total n=33  Frail older people in a residential care facility.  Mean age: total 83.8 (8.0; 63-98); Tai Chi 81.1 (8.0); Yoga 84.9 (6.7); UC 85.4 (9.1) years.  % female: total 72.7%; Tai Chi 72.7%; Yoga 90.9%; UC 54.5%. | Modified tai chi program (2 x week for 14 weeks). 30min sessions conducted by experienced and certified tai chi instructors.  CG: **Yoga intervention**. 2 x per week, 30 mins for 14 weeks. **Usual care (UC).** Including Stay Active program (seated exercise sessions), physical culture (games and group activities) and gym activities (bikes, pulleys, massage). | **Physical function:** BBS (baseline and post-intervention). **QOL**: Dementia quality of life (DQOL) questionnaire (baseline, mid-intervention (7 weeks) and post-intervention). | **Physical function**: no significant between group difference on the BBS. **QOL**: no significant between group difference on the DQOL.  **Adherence**: The average attendance for the entire intervention period was greater for the tai chi group (78.2%) than the yoga group (76.3%). **Adverse events:** one adverse event occurred during the study period; a participant in the yoga group experienced a fall. |
| (49) | n= 40 (20 in each group).  Analysed total n=40.  Nonagenarians living in a geriatric nursing home.  Mean age: IG 92 (2); CG 92 (2) years.  % female: IG 80%; CG 80%. | IG: Muscle strength training program (3 x week for 8 weeks). Trained at 30-70% of 1-RM; 2-3 sets of 8-10 repetitions. Each session lasted approx. 45-50mins and was supervised by a qualified fitness specialist.  CG: Usual care group. Instructed on the positive effects of regular exercise. They performed mobility exercises for 40 to 45 minutes per day, 5 x week. | **Physical function**: 8-m walk test and 4-step (20-cm height each) stairs test, TUG 3-m (baseline, 8 weeks (end of intervention), 12 weeks (end of detraining). | **Physical function**: no significant group by time interactions were found for the 8-m walk test, 4-step stair test or TUG test.  **Adherence**: adherence to training in the IG averaged 74%. **Adverse event**s: no major adverse events attributable to intervention. Some participants complained of mild muscle pain associated with the leg press exercises. |
| (50) | n=15; IG n=8; CG n=7.  Analysed total n=13 (IG n=7; CG n=6).  Nursing home residents with low physical performance.  Mean age: total 84.0 (7.4, 74-98); IG 84.4 (6.3); CG 83.6 (8.9) years.  % female: total 80%; IG 88%; CG 71%. | IG: Progressive Whole Body Vibration training (2 x week for 10 weeks) with exercises. Training supervised by a physiotherapist.  CG: Sham training. Same training as WBV group but without vibrations. | **Physical function:** SPPB, TUG 3m, 4m walking speed (baseline and 10 weeks). | **Physical function**: no significant between group difference in SPPB score, walking speed (normal and fast m/s) or TUG 3m.  **Adherence**: average attendance for the WBV and sham training was 74% and 73%, respectively. **Acceptability**: the high 18 Hz vibration frequency, and 26 Hz, was poorly tolerated and had to be mostly replaced by the low 12 Hz frequency. |
| (51) | n=159; WBV + exercise n=81; exercise n=78.  Analysed total n=159.  Institutionalised elderly people residing in nursing homes.  Mean age: total 82; WBV + exercise 82.3 (7.7); exercise 82.5 (7.1) years.  % female: total 67.2%; WBV + exercise 65.4%; exercise 69.2%. | IG: WBV + exercise program (3 x week for 6 weeks). Performed static/dynamic exercise (balance and strength training) individually on a vibratory platform. Sessions were 30mins and performed in small groups (2-8 people).  CG: Exercise group (3 x week for 6 weeks). Performed the same static/dynamic exercises but no WBV was imposed. Sessions were 30mins and performed in small groups (2-8 people). | **Physical function:** Tinetti test, TUG 3m, Sit-to-Stand (STS) test (baseline, post-intervention (6 weeks), and 6 months after the study). | **Physical function:** no significant difference between groups at either 6 weeks or 6 months on Tinetti total score, TUG or STS test.  **Adverse events:** no severe adverse effects were observed in relation to the intervention. Pain (18.25%) in the knee and lumbar spine, and soreness (12.6%) in quadricipital and gastrocnemius muscles were reported. Itching (0.6%), erythema (1.2%), and oedema (0.6) of the legs were also reported. |
| (74) | n=28; IG n=14; CG n=14.  Analysed total n=26 (IG n=12; CG n=14).  Residents of a geriatric nursing home.  Mean age: IG 85.8 (7.8); CG 85.7 (3.9) years.  % female: total 85.7%. | IG: Individual exercise group (2 x week for 16 weeks). Received individual physical therapy for 20min, consisting of resistance training for the lower extremities, balance exercises and indoor gait exercise; the choice of therapy was based on each participant's needs.  CG: Group exercise intervention. Individual physical therapy 2 x per week for 20 min between month 1 and 2. After month 2, individual physical therapy was conducted 1 x per week, and also engaged in 20min group walk around facilities walking trail in small groups with a physical therapist | **Physical function**: Barthel Index, gait velocity 14m, step length (baseline, once per month for 4 months). **QOL**: Life Satisfaction Index (LSIA, quality of life) (baseline, once per month for 4 months). **Mental health**: Philadelphia Geriatric Centre Morale Scale (PGC) (baseline, once per month for 4 months). | **Physical function:** no significant between group differences on Barthel Index, gait velocity or step length. **QOL**: no significant between group difference on LSIA. **Mental health:** No significant between group difference on PGC. |
| (52) | n=170; IG n=87; CG n=83.  Analysed total n=154 (IG n=77; CG n=77).  Nursing home residents with dementia.  Mean age: total 86.9 (7.4); IG 87.3 (7.0); CG 86.5 (7.7).  % female: total 73.5%; IG 72.4%; CG 74.7%. | IG: High Intensity Functional Exercises program (2 x week for 12 weeks). Included strength and balance exercises; individually tailored, instructed and supervised. The exercise sessions lasted 50–60 min, completed in small groups with physiotherapists.  CG: control activities were light physical activity in sitting, reading, playing games, listening to music and making conversations. 2 x per week for 50-60mins led by occupational therapists, nursing staff, volunteers or activity-leader. | **Physical function:** BBS, 6m walking test, 30-s chair stand test, Barthel Index (baseline, 12 weeks (intervention completion) and 6 months). **QOL**: Quality of life in late stage dementia scale (QUALID) (baseline, 12 weeks (intervention completion) and 6 months). **Mental health:** Cornell Scale for Depression in Dementia (baseline, 12 weeks (intervention completion) and 6 months). | **Physical function:** from baseline to 6 months follow-up, the IG improved their BBS score while the CG declined and the difference between the groups was significant (p = 0.031). No between group difference for changes on the 6m walking test, chair stand test or Barthel Index. **QOL**: no between group difference for changes on the QUALID. **Mental health**: no between group difference for changes on the Cornell Scale.  **Adherence**: attendance rates of the IG and CG were 75% and 69%, respectively. **Adverse events**: no adverse effects of exercise were observed. |
| (53) | n=170; IG n=87; CG n=83.  Analysed total n=163 (IG n=82; CG n=81).  Nursing home residents with dementia.  Mean age: total 86.7 (7.4); IG 86.9 (7); CG 86.4 (7.8) years.  % female: total 73.6%; IG 72%; CG 75.3%. | IG: High Intensity Functional Exercises program (2 x week for 12 weeks). Included strength and balance exercises; individually tailored, instructed and supervised. The exercise sessions lasted 50–60 mins, completed in small groups with physiotherapists.  CG: activities included light physical activity in sitting, reading, playing games, listening to music, and making conversations. 2 x per week for 50-60mins led by occupational therapists, nursing staff, volunteers or activity-leader. | **Physical function:** BBS, 6m walking test, 30-s chair stand test, Barthel Index (baseline and post-intervention). **QOL**: Quality of life in late stage dementia scale (QUALID) (baseline and post-intervention). **Mental health:** Cornell Scale for Depression in Dementia (baseline and post-intervention). | **Physical function:** from baseline to 12 weeks, the IG improved their BBS score more than the CG, difference in change between groups was significant (p = 0.02). Significant between group difference for change in ADLs on Barthel Index (p<0.01), IG slight improvement and decline in CG. No between group difference for changes on the 6m walking test or chair stand test. **QOL**: No between group difference for changes on the QUALID. **Mental health:** No between group difference for changes on the Cornell Scale.  **Adherence**: Attendance rates of the IG and CG were 75% and 69%, respectively. **Adverse events**: no adverse effects of exercise were observed. |
| (54) | n=186 (93 in each group).  Analysed total n=186.  Older adults with dementia living in residential care facilities.  Mean age: total 85.1 (7.1); IG 84.4 (6.2); CG 85.9 (7.8) years.  % female: total 75.8%; IG 75.3%; CG 76.3%. | IG: High Intensity functional exercise program (5 x fortnight for 16 weeks). Includes lower limb strength and balance exercises. Sessions were conducted in small groups for 45mins and supervised by physical therapists.  CG: Seated control activity (e.g. listening to music or readings). Supervised by occupational therapist or occupational therapy assistant. | **Physical function:** Functional Independence Measure (FIM), Barthel Index, Berg Balance Scale (BBS) (baseline, 4 and 7 months). | **Physical function:** no significant between group difference on the BI or FIM at 4 or 7 months. Significant between group difference on the BBS at 4 months (but not 7); BBS improved for the IG and declined for the CG at 4 months (p<.001). **Subgroup analyses according to dementia type; Alzheimer’s versus other (non-Alzheimer’s) dementia. The effect of exercise was significant in favour of participants with non-Alzheimer's dementia at 4 (p=.04) and 7 months (p=.01) for BBS, and at 7 months for FIM (p=.02) and BI scores (p=.01); significant positive effects for those with non-Alzheimer's dementia. FIM and BBS scores reflected negative effects in participants with Alzheimer’s disease at 7 months.  **Subgroup analyses according to cognition level. Participants with higher cognitive levels benefitted more from exercise than those with lower cognitive levels for BBS at 7 months only. Negative effect for BBS in participants with lower cognitive function at 7 months  **Adherence**: adherence rates of the IG and CG were 73% and 70%, respectively. **Adverse events:** all reported adverse events related to exercise sessions were minor or temporary. |
| (55) | n=55; MFT n=18; MFT+ Balance n=18; self-administered training n=19.  Analysed total n=48 (MFT n=16; MFT+ Balance n=14; self-administered training n=18).  Older adults residing in an institutional setting.  Mean age: MFT 84.7 (5.5); MFT+ Balance 85.0 (4.2); self-administered training 86.1 (7.3) years.  % female: MFT 66.6%; MFT+ Balance 88.8%; self-administered training 73.6%. | IG: Muscle force training **(MFT)** (2 x week for 13 weeks). Sessions were 60mins and took place under the supervision of physiotherapists.  CG: **MFT+ Balance.** Training took place under supervision of a physiotherapist, 60 mins, 2 sessions per week for 13 weeks. **Self-administered training**. Included exercises in the sitting position and standing position.  Supervised by geriatric nurses, 60 mins, 2 sessions per week for 13 weeks. | **QOL**: 15D QOL (baseline, 3 and 6 months). | **QOL**: no significant between group difference on 15D QOL at 3 or 6 months. |
| (56) | n=35; IG n=18; CG n=17.  Analysed total n=20 (IG n=11; CG n=9).  Older female nursing home residents.  Mean age: total 84.3 (7.9); IG 84.4 (7.7); CG (88.9 (5.3) years.  % female: 100%. | IG: Progressive elastic resistance training (ERT) program of moderate intensity (3 x week for 12 weeks).  CG: Did not receive any placebo or treatment. | **Physical function:** SPPB (baseline and post-intervention). | **Physical function**: significant group-time interaction for the SPPB score (p <.01, partial η2 = 0.36], gait speed (p = .04, partial η2 = 0.22) and chair rise (p < .01, partial η2 = 0.53). In the IG, the SPPB score (p =.03) was significantly improved at post-training compared to pre-training values, no significant change in CG. Gait speed (p<.05) and chair rise (p<.01) performance also significantly improved in the IG, no significant changes in CG. |
| (57) | n=162; IG n=79; CG n=83.  Analysed total n=162  Older adults permanently living in a nursing home.  Mean age: total 83.1 (7.9); IG 83.3 (8.2); CG 82.8 (7.8) years.  % female: total 92%; IG 91.6%; CG 92.4%. | IG: Dance-based exercise program (EXDASE) (1 x week for 12 weeks).  CG: Waitlist control. | **Mental health:** GDS-15 (baseline and post-intervention). | **Mental health**: significant group-by-test interaction for GDS (p=.001). At 3 months GDS score in the IG had significantly improved (p=.005). CG experienced further worsening of depressive symptoms, although the difference was not statistically significant.  **Adherence**: the average attendance rate was 84.6%, and all participants completed more than 50% of the classes. **Adverse events:** there were no reported injuries. |
| (58) | n=68; Group A n=27; Group B n=26; Group C n=15.  Analysed total n=68.  Elderly people with mild cognitive impairment residing in residential care homes.  Mean age: total 78.3 (9.5); Group A 79.2 (10.0); Group B 76.4 (11.3); Group C 79.4 (6.7) years.  % female: total 56.2%. | IG: Aerobic exercise program **(Group A)** (3 x week for 12 weeks). Aerobic exercise (cycling in a recumbent bike) at 40% of heart rate. 30 min sessions supervised by a physiotherapist.  CG: **Group B.** Same aerobic exercise program except that it was set at 60% of participant's heart rate. **Group C**. Recreational activities (playing cards, reading newspapers, handicrafts). | **Physical function:** TUG 3m (baseline, post-intervention, follow-up (6 months since intervention started)). | **Physical function**: no significant differences were found on the TUG among the three groups at any timepoint. |
| (59) | n=74; IG n=25; CG n=49.  Analysed total n=74.  Institutionalized older adult without cognitive impairment living in long term home care.  Mean age: IG 77.9 (8.7); CG 83.5 (7.0) years.  % female: IG 47.0; CG 31.8%. | IG: Encouraged to cycle continuously (daily for 60 weeks) on a recumbent bike at their self-selected intensity at least for 15 min every day. A physiotherapist monitored the sessions.  CG: Usual recreational activities (e.g. playing cards, playing board games), daily (60mins), performed freely and without supervision. | **Physical function**: Katz index functional independence, TUG 3m (baseline and post-intervention). | **Physical function**: no significant between group differences were found for changes on the Katz Index or TUG. |
| (60) | n=30 (n=15 in each group).  Analysed total n=23 (IG n=12; CG n=11).  Frail women residing in a geriatric institution.  Mean age: IG 83.3 (6.7); CG 84.1 (5.8) years.  % female: 100%. | IG: Strength circuit training exercise schedule (3 x week for 12 weeks). Exercises sessions were 45 min performed under the supervision of a kinesiologist in small groups while sitting in wheelchairs.  CG: Usual behaviour. | **Physical function**: Barthel Index (baseline and post-intervention). | **Physical function:** significant difference between groups for the Barthel Index score (p<.0001). The IG showed a significant improvement in the Barthel Index score after the training, CG maintained a similar score.  **Adherence**: the IG attended 74.8% of the 36 scheduled sessions. **Adverse events:** no adverse events related to the exercise program were observed. |
| (63) | n=24 (n=12 in each group)  Analysed total n=21 (IG n=11; CG n=10)  Elderly nursing home residents in the later stages of Alzheimer’s disease.  Mean age: IG 83(6); CG 85 (6) years.  % female: 100%. | IG: Simple aerobic walking activity (4 x week for 24 weeks). 30 mins of moderate-intensity walking with a caregiver (partners or adult relatives who habitually frequented the NH).  CG: Participated in the daily organized (e.g. bingo, patchwork sewing). | **Physical function:** Barthel Index, 6WT (baseline and post-intervention). | **Physical function:** significant group by time interaction for the Barthel Index (p=.003). IG showed significant improvement in Barthel index scores while the CG maintained the same scores. Significant group by time interaction for 6WT (p<.001). IG showed significant improvement in performance on the 6WT, performance declined significantly in CG.  **Adherence**: the walking group attended 93.4% of the scheduled training sessions. **Adverse events**: no adverse events related to the exercise program. |
| (79) | n=117; WBV n=36; SIM n=35; CG n=46.  Analysed total n=117.  Frail elderly individuals living in residential care facilities.  Mean age: total 82.4 (7.9); WBV 79.4 (1.1); SIM 83.7 (1.2); CG 84.3 (1.3) years.  % female: total 64.9%; WBV 58.3%; SIM 77.1%; CG 60.8%. | IG: WBV exercise group (3 x week for 16 weeks). Supervised by research staff and trained students.  CG: Simulated WBV **(SIM).** Mimicked WBV stance and duration. 3 x per week for 16 weeks. **Control group.** | **Physical function:** TUG, Parallel Walk Test (PWT), 10-m Timed Walk (10mTW) test, Barthel Index (baseline, 8 weeks, 16 weeks of the training intervention and 3, 6- and 12-months post-intervention). | **Physical function**: significant difference between WBV and SIM, and WBV and CG on TUG at 8 weeks (p=0.18, p=0.16), and 16 weeks (p=0.11, p=0.00). Significant difference between SIM and CG at 12 months (p=.007). Significant difference between WBV and SIM on 10mTW at 3 months (p=.005). No other between group differences on 10mTW at 8weeks, 16 weeks, 6 or 12 months.  Significant difference between WBV and SIM, and WBV and CG on PWT time at 8 weeks (p=.006, p=.009) and 16 weeks (p=.006, p=.007). Significant difference at 3 months between WBV and SIM (p=.003). Significant difference at 6 months (p=.040) and 12 months (p=.034) between WBV and CG. Functional test performance improved for WBV across the intervention period, in particular, improvements of 7.3% for TUG and 14.9% for PWT, and declined in non-WBV groups. Significant between group difference on Barthel Index between WBV and CG at 8 weeks (p=.026), 16 weeks (p=.000), 3 months (p=.007), 6 months (p=.002) and 12 months (p=.006) .  After 16 weeks of training, Barthel Index scores were on average 5.8% higher than baseline for the WBV group (P<0.001), compared to -0.5% and -6.4% lower for SIM and CG (p=.003).  **Adherence**: WBV and SIM groups attended 93% and 89% of sessions, respectively. **Acceptability**: Participants frequently described WBV exercise as fun to use and expressed enjoyment. |
| (76) | n=60 (n=30 in each group).  Analysed total n=60.  Older adults with mild cognitive impairment residing in a nursing home.  Mean age: IG 66.7 (5.4); CG 65.2 (4.6) years.  % female: IG 30%; CG 36.7%. | IG: Handball training (5 x week for 24 weeks). Conducted by physiotherapists and nurses for 30mins.  CG: Usual activities. | **Physical function**: ADL scale (baseline, 3 and 6 months). | **Physical function:** significant group-time interaction for ADLs (p=0.000). ADL score improved in the IG after 3 and 6 months (p<0.05) while there were no significant changes in CG. |
| (61) | n=204; BE n=51; BE+VS n=51; FET n=51; FET+VS n=51  Analysed total n=204  Older people who are living a sedentary lifestyle in nursing homes.  Mean age: BE 74.3 (8.3); BE+VS 73.2 (7.3); FET 74.8 (7.4); FET+VS 73.7 (7.5) years.  % female: BE 62.7%; BE+VS 56.8%; FET 56.8%; FET+VS 54.9%. | IG: Basic exercises without verbal stimulation **(BE)** (2 x week for 12 weeks). Included exercises performed in sitting position; contained elements of aerobic fitness and stretching. Program conducted in small groups for 30mins by a physiotherapist.  Basic exercises with verbal stimulation **(BE+VS**). Same exercise program as BE group but also incorporates verbal stimulation. Function exercise training without verbal stimulation **(FET).** Strengthening and stretching, exercises performed in a sitting position using a Thera-Band and gymnastic sticks. Sessions performed by physiotherapists in small groups 2 x per week for 30mins, 12 weeks. **Functional exercise training with verbal stimulation (FET+VS).** Same exercise as functional exercise training group but with verbal stimulation. | **Physical function**: SPPB, Physical Activity Scale for the Elderly, ADL-IADL scale, TUG 3m, 10m walk test, BBS (baseline, post-intervention and 24 weeks). **QOL**: SF-36 questionnaire (baseline, post-intervention and 24 weeks). | **Physical function**: after 12 weeks of exercises, there was a statistically significant difference between the BE and FET + VS groups on SPPB (p<.001), IADL (p=0.04), 5x STS (p=0.01), TUG (p=0.03), gait speed (p=0.001), BBS (p<.001). In the FET + VS group, SPPB, IADL, 5x STS, TUG, gait speed and balance significantly improved, in comparison to the BE group.  After 24 weeks, the greatest effects were noted in the FET + VS group, in comparison to the BE group in SPPB (p<.001), ADL (p<.001), IADL (p<.001), 5x STS (p<.001), TUG (p<.001), gait speed (p<.001), BBS (p<.001), total physical activity (p=0.009) and leisure time activity (p=0.019). At 24 weeks, in the FET + VS group, compared to the other exercising groups (BE, BE + VS, FET), there were statistically significant, larger positive changes in SPPD, leisure-time activity, performance of complex daily activities, TUG and gait speed (p < 0.001).  **QOL**: After 12 weeks, there was a statistically significant difference between the BE and FET + VS groups on QOL (physical component) (p<.001). In the FET + VS group, QOL (physical component) significantly improved, in comparison to the BE group.  At 24 weeks, in the FET + VS group, compared to the other exercising groups (BE, BE + VS, FET), there were statistically significant, larger positive changes in QOL (physical component) (p < 0.001). No significant between group differences at 12 or 24 weeks on mental health component of QOL. |
| (62) | n=21; VR n=10; conventional exercise n=11.  Analysed total n=18 (VR n=7; conventional exercise n=11).  Older adults in a nursing home.  Mean age: VR 70.1 (4.0); conventional exercise 73.1 (4.5) years.  % female: VR 42.9%; conventional exercise 81.8%. | IG: Virtual reality group (VR) (3 x week for 6 weeks). Balance training with the BTS NIRVANA VR Interactive System under the supervision of a physiotherapist.  CG: Conventional balance exercises. 3 sessions per week for 6 weeks. Conducted under the supervision of a researcher. | **Physical function:** BBS, TUG 3m (baseline and post-intervention). | **Physical function:** no significant group-by-time interactions for BBS or TUG.  **Adherence**: the participation rate was 96% in VR group and 87% in conventional exercise group. **Adverse events:** no serious balance loss/falling, pain or injury was recorded during the study. |

6MWT 6-minute walk test; ADL Activities of Daily Living; BBS Berg Balance Scale; BI Barthel Index; CG Comparator Group; COVS Clinical Outcome Variable Scale; CRT Chair rise test; FIM Functional Independence Measure; FR Functional Reach test; FTSTS 5 x sit to stand test; GDS Geriatric Depression Scale; IADL Instrumental activities of daily life; IG Intervention Group; NHLSD Nursing Home Life Space Diameter; NHPPT Nursing Home Physical Performance Test; PA Physical activity; PGC Philadelphia Geriatric Centre Morale Scale; POMA Performance Oriented Mobility Assessment; POMS-SF Profile of Mood States Short Form; PWT Parallel Walk Test; QOL Quality of Life; SD Standard Deviation; SF-36 Short form 36; SFT Senior fitness test; SOF Study Osteoporotic Fractures Index; SPPB Short Physical Performance Battery; STS Sit to stand test; SWS Satisfaction with Life Scale; TT Tinetti test; TUG Timed Up and Go; WBV Whole Body Vibration
